# From Brittle to Ultraductile: Precise Ionic PEG with Dynamic Networks and Reconfigurable Crystallites

**DOI:** 10.1021/jacsau.6c00959

**Published:** 2026-08-27

**Authors:** Jie Xu, Wenlin Zhang, Hung-Jue Sue, Shuyi Xie

**Affiliations:** † Department of Chemical Engineering, 14736 Texas A&M University, College Station, Texas 77843, United States; ‡ Department of Chemistry, 3728 Dartmouth College, Hanover, New Hampshire 03755, United States; § Polymer Technology Consortia, Department of Materials Science and Engineering, Texas A&M University, College Station, Texas 77843, United States

**Keywords:** semicrystalline polymers, ionic associations, ultraductile thermoplastics

## Abstract

Achieving high ductility and toughness in semicrystalline polymers without sacrificing stiffness remains a longstanding challenge. Here we show that sparse precise ionic functionalization can fundamentally alter the mechanical behavior of a semicrystalline polymer. Ionic poly­(ethylene glycol) (iPEGs) containing precisely spaced imidazolium groups (∼1.5 mol %, every 62 repeat units) undergo a striking transition from brittle to ultraductile behavior, which exhibits stable neck propagation and apparent strain hardening at moderate molecular weight of 41 kDa. Tensile behavior, melt rheology, and X-ray scattering spectra reveal that this highly ductile behavior arises from a coupled ionic-semicrystalline architecture. Dynamic ionic associations introduce long-lived transient constraints that enhance the amorphous phase resistance to deformation and sustain load-bearing connectivity between crystallites, while ionic incorporation suppresses lamellar thickening and produces thin PEG crystallites capable of deformation-induced reorganization. The resulting interplay between crystallite connectivity, tie-chain load transfer, and reconfigurable crystallites enable stable neck propagation rather than brittle fracture. These results establish sparse ionic “stickers” as a molecular design principle for strengthening crystal-mobile semicrystalline polymers without introducing additional phases or permanent covalent cross-links.

## Graphical abstract

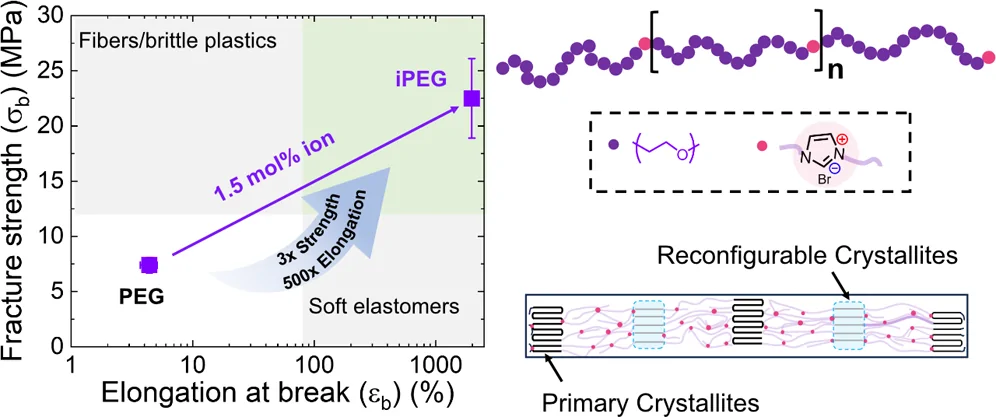

## 1 Introduction

Semicrystalline polymers rarely possess high strength with large ductility. In these materials, crystallinity generally improves dimensional stability but restricts molecular mobility and energy dissipation, leading to reduced ductility, slow stress relaxation, limited transport, and poor recovery after deformation.^1–9^ This intrinsic trade-off between strength and ductility represents a long-standing challenge in polymer physics and restricts the use of many sustainable polymers in load-bearing applications. A few commodity polyolefins, most notably polyethylene (PE), due to the favorable structural and dynamical properties in the semicrystalline state, exhibit stable neck propagation and strain-hardening to high draw ratios.^5,10–14^ However, many technologically important crystallizable polymers, such as polyethers, polyesters and polyamides, are stiff but comparatively brittle. Their toughness (*U*_T_) is typically enhanced through rubber toughening, copolymerization, blending, or inorganic fillers that introduce additional phases which complicate recycling.^15–20^ A simple, molecular-level design strategy to transform a brittle, crystal-mobile semicrystalline homopolymer into an ultraductile, energy-dissipating thermoplastic without architectural complexity or permanent cross-links is still lacking.

Dynamic and reversible interactions have recently been explored to reconcile stiffness, toughness, and reprocessability in polymer networks, but almost all efforts involve only amorphous or weakly crystalline matrices. In these systems, supramolecular motifs (e.g., hydrogen bonds, π–π stacking, metal–ligand complexes, ionic associations) act as physical cross-links that break and reform under load, dissipating energy without catastrophic fracture.^21,22^ For example, densely packed urethane hydrogen-bond arrays in segmented poly­(urethane-urea)­s yield spider-silk-like elastomer combinations of modulus, strength, and toughness,^23^ while high-density Fe-carboxylate ionic clusters can convert a soft self-healing network into a much tougher, stretchable elastomer.^24^ Likewise, dynamic ionic “stickers” based on alkylimidazolium groups and their counterions greatly increase modulus and toughness.^25^ Related concepts have also enabled tough ionogels (polymer networks swollen in ionic liquids), in which reversible ionic associations act as physical cross-links that dissipate energy under load.^26,27^ Collectively, these studies show that secondary interactions, like ionic associations, are robust, reconfigurable cross-links for strengthening polymers. Beyond amorphous networks, precisely sequenced PE bearing periodically placed acids or ions have shown that associative ionic domains can form well-defined nanostructures and reorganize under tensile deformation, as captured by in situ X-ray scattering and linked to strain hardening.^28–32^ However, these insights have been established largely in nonpolar polyolefin backbones, where the segregation of ionic/acidic motifs from polymer backbone is substantial. Moreover, the relatively high ionic content (>10 mol %) significantly disrupts the intrinsic crystallinity of PE, which decreases to zero at an ionic content of 13 mol %.^28–30^

We hypothesize that at low ionic content (<2 mol %) and with good compatibility between the ions and the polymer backbone, periodic placement of ionic sites can couple dynamic amorphous phase reinforcement with reconfigurable semicrystalline microstructure to enable synergistic enhancement of ductility without sacrificing stiffness. Here, a simple but effective strategy is presented to engineer a brittle and highly crystalline polymer (*X*_c_ > 70%) into an ultraductile plastic with modest crystallinity (*X*_c_ ≈ 30%) by introducing 1.5 mol % of ionic group periodically on a polar polymer backbone. Note that no nanoscale ion cluster is anticipated in this system, unlike conventional ionomers.^30,33,34^ Instead, the sparse chain-bound ionic groups are expected to regulate amorphous-phase dynamics while maintaining intercrystallite connectivity, thereby overcoming the conventional trade-off between strength and ductility.^21,22^ Beyond enhancing ductility, such dynamic ionic/crystalline coupling may also allow thermally triggered relaxation of deformation-induced orientation.

Poly­(ethylene glycol) (PEG) offers a stringent test of this design principle. Given PEG’s wide use in biomedical devices,^35–37^ drug delivery,^37–39^ polymer electrolytes^40–42^ and surfactants,^43^ its intrinsic brittleness in the neat semicrystalline state is a persistent limitation. High-molecular-weight linear PEG is a “classical” semicrystalline polymer: it forms thick lamellae crystals (*d*_c_ ≈ 15–60 nm) with high crystallinity (*X*_c_ > 70%),^14,44–46^ in which chains predominantly fold into the same lamellae, an energetically and entropically favored pathway.^46–49^ This leaves only thin amorphous layers (*d*_a_ ≈ 2–8 nm), which limits the deformability.^14,44–46,50^ These impart superior stiffness (Young’s modulus, *E* ≈ 100–200 MPa) but limited tensile extensibility (ε_b_ ≈ 4–80%) in the neat semicrystalline state.^51^ At the same time, PEG crystals display high intracrystalline α-relaxation (α_c_) mobility, originating from defect-mediated chain diffusion along the crystal axis.^45,50,52^ Such α_c_-mobility could in principle support large plastic deformation if the crystalline morphology and tie-chain network were appropriately tuned.^8,44,52^ In conventional high-MW PEG homopolymers, however, this intrinsic crystal mobility is largely “wasted” in thick, space-filling lamellae.^2,52^

Here, we design a family of ion-containing PEGs in which ionic groups are periodically introduced along the backbone, approximately every 62 EO repeat units. Such sparsely charged polymers, with cations directly tethered to the backbone, are classified as “ionenes”.^31,53–56^ We synthesized PEG ionenes (iPEGs) via solvent-free Menshutkin step-growth polymerization from PEG oligomer precursors (*M*_n_ = 2.8 kDa, *N* = 62, *D̵* = 1.03) with both ends functionalized into imidazole and bromo groups, respectively (Scheme 1).^31,57^ This architecture ensures that the ionic groups are uniformly distributed along the chains, yet sparse enough (<1.5 mol %) that PEG still crystallizes. By deliberately varying the oligomer stoichiometry, we access iPEGs with controlled molecular weights (named as iPEG_*M*_n_) at fixed ionic spacing, and compare their tensile behavior, melt rheology, and semicrystalline morphology (DSC and SAXS/WAXS) to a neutral diol-PEG benchmark (*M*_v_ = 100 kDa, named as HO–PEG–OH_100k). Beyond its role as a model crystal-mobile polymer, such ionically modified PEG also offers opportunities in applications where the intrinsic properties of PEG are advantageous, such as stretchable polymer electrolytes, biomedical materials, soft actuators, and separation membranes.

**Scheme 1:**
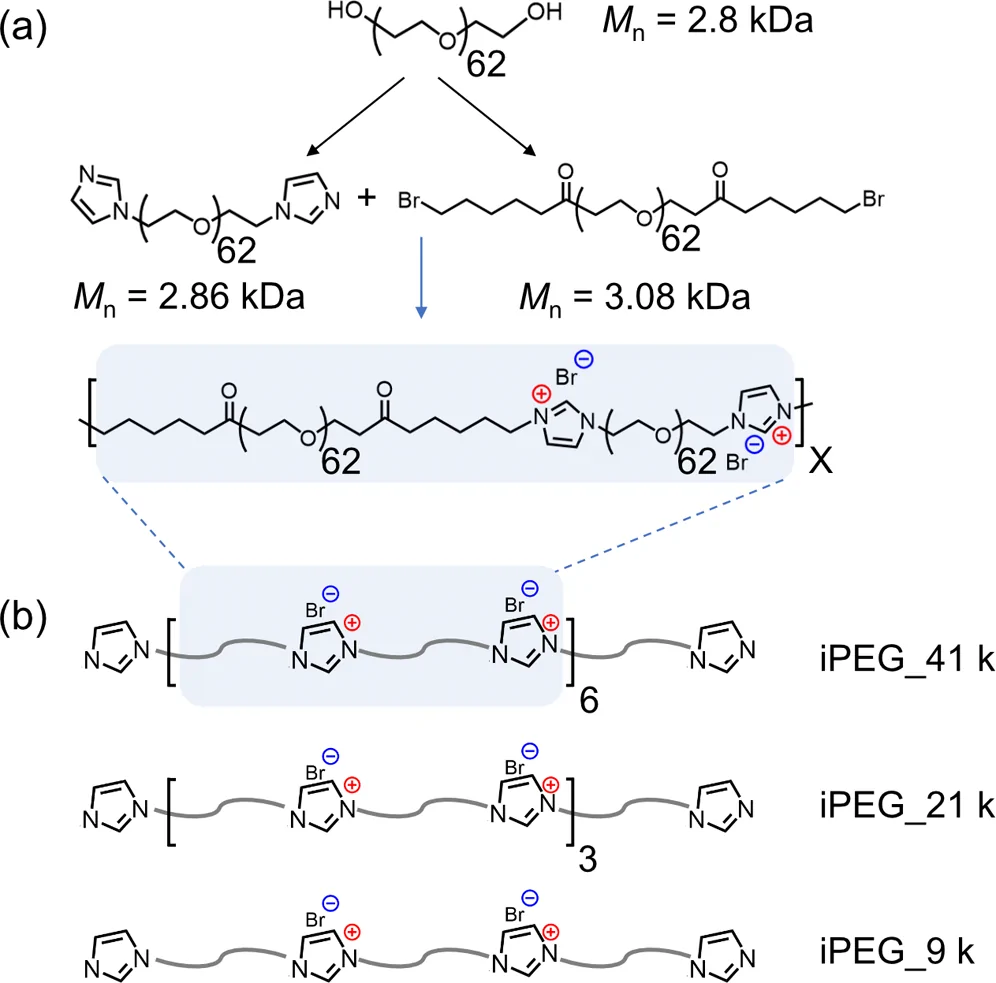
(a) Synthesis Routes and (b) a Library of iPEGs with a Fixed Ion–Ion Spacing (*N* = 62) but Different Total Molecular Weights (9, 21, and 41 kDa)

## 2 Results and Discussion

### 2.1 iPEG Synthesis

We selected an unentangled diol PEG (*M*_n_ = 2800 g/mol, *D̵* ≈ 1.03) as the precursor. Following the established procedures,^58–61^ diol PEG was first reacted with methanesulfonyl chloride and then with imidazole to yield di-imidazole PEG with high chain-end fidelity (Figure S1). In a separate route, diol PEG was reacted with 6-bromohexanoyl chloride to obtain dibromo PEG with high chain-end fidelity (Figure S2).^53,62–64^ By adjusting the stoichiometric ratio of the di-imidazole and dibromo oligomers, we accessed a series of linear iPEGs with controlled number-average molecular weights (*M*_n_ ≈ 9, 21, and 41 kDa) at fixed ionic spacing (*N* = 62) via the solvent-free Menshutkin reaction (Scheme 1 and Table 1).^31,57,65–67^ Under these step-growth conditions, all oligomers are completely consumed to give well-defined chains whose chain-end functionalities are identical and *M*_n_ cannot increase during a postcharacterization process.^65^ In our system, an excess of di-imidazole led to iPEG bearing imidazole end groups. The expected polymer structure and end groups were confirmed by ^1^H NMR spectroscopy (Figure S3) and dynamic light scattering (DLS) (Table 1 and Figure S4). Thermogravimetric analysis (TGA) confirmed that the water contents of all samples were below 2% (Figure S9); therefore, atmospheric moisture was not considered a significant factor in the subsequent measurements.

**Table 1:** iPEGs with Precise Ion Distributions and Different Molecular Weights

sample ID	monomer molar ratio^a^	theoretical *M*_n_ (g/mol)^b^	experiment *M*_n_ (g/mol)	ionic density (mol %)^e^
iPEG_41k	1.00:1.00	/	41,300^c^	1.5
iPEG_21k	1.0:0.75	21,000	22,800^c^	1.4
iPEG_9k	1.0:0.50	9000	9000^b^ ^,^ ^d^	1.1

a Di-imidazole PEG:dibromo PEG.

b Calculated molecular weights determined by Carothers equation.^14,68^

c Molecular weights are determined by DLS.

d Used as the reference to determine the *M*_n_ of other ionenes by DLS

e Ionic density calculated as mol % of ionic repeat units relative to EO repeat units.

### 2.2 Macroscopic Mechanical Response of iPEGs and Working Hypothesis

Tensile engineering stress–strain curves revealed outstanding tensile performance for iPEG_41k. As shown in Figure 1 and Table 2, iPEG_41k is an ultraductile, tough thermoplastic: it combines *E* = 230 ± 30 MPa, ε_b_ = 1950 ± 190%, σ_b_ = 22.4 ± 3.1 MPa and *U*_T_ = 311.0 ± 66.3 MJ/m^3^. The relatively large standard deviation in toughness for iPEG_41k likely arises from the intrinsic variability of highly extensible semicrystalline polymers, including necking instability and variations in neck propagation. Its deformation profile shows the classic high-density polyethylene (HDPE)-like sequence of yield, stable neck propagation, and strain-hardening. In contrast to HO–PEG–OH_100k, which fails in a brittle manner, the iPEG_41k sample displays a significant ductile behavior, placing its performance on par with or exceeding polyethylene or ultrahigh MW PEGs. The pronounced stress serrations (cyclic stress drops and recoveries) observed in iPEG_41k in Figure 1a are particularly notable and suggest intermittent structural reorganization events during deformation (discussed in detail in Section 2.4.2). This result reveals that introducing only ∼1.5 mol % of well-defined ionic centers into the PEG backbone fundamentally changes the PEG mechanical behavior, transforming it from brittle crystals into a superductile, strong thermoplastic.

**Figure 1:**
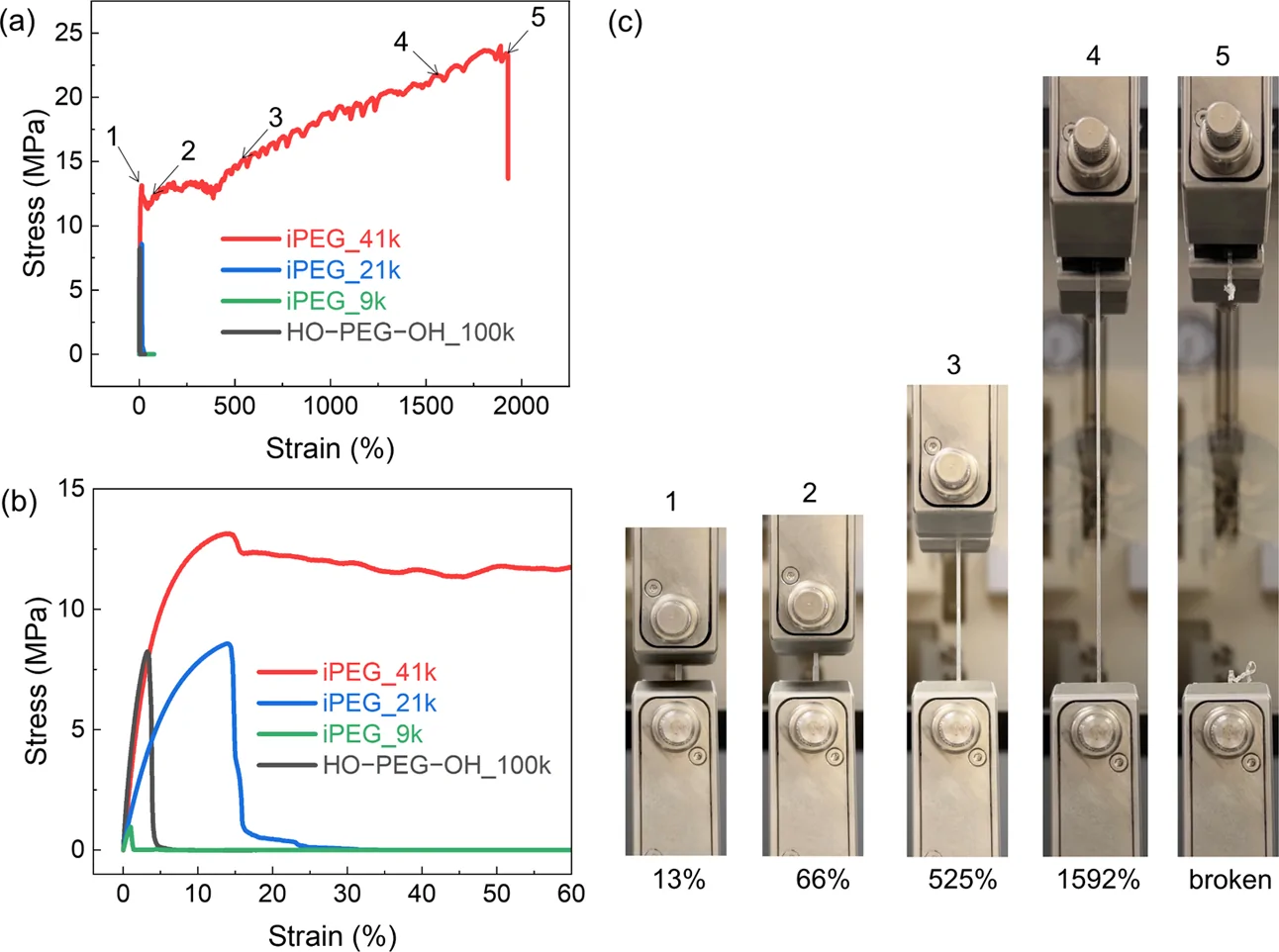
Mechanical characterization of iPEGs with fixed ionic spacing and varying molecular weight, compared with HO–PEG–OH: (a,b) tensile stress–strain curves reveal a chain-length threshold for stable necking and strain hardening; (c) photographs of iPEG_41k during tensile testing at selected strains.

**Table 2:** Mechanical Properties of iPEGs and HO–PEG–OH_100k (room Temperature)

sample ID	Young’s modulus (MPa)^a^	elongation at break (%)	stress at break (MPa)	toughness (MJ/m^3^)^b^
iPEG_41k	230 ± 30	1950 ± 190	22.4 ± 3.1	311.0 ± 66.3
iPEG_41k_q	160 ± 10	2430 ± 170	21.4 ± 0.9	342.1 ± 21.4
iPEG_21k	130 ± 20	13 ± 1	8.6 ± 1.1	0.9 ± 0.1
iPEG_9k	120 ± 20	0.9 ± 0.2	0.8 ± 0.2	0.005 ± 0.002
HO–PEG–OH_100k	240 ± 50	4.4 ± 0.7	7.4 ± 0.7	0.26 ± 0.06
HO–PEG–OH_100k/salt	1 ± 0.6	26 ± 5	0.1 ± 0.03	0.02 ± 0.01

a Calculated from the slope of the linear range of the stress–strain curve.

b Calculated by integrating of the entire stress–strain curve.

This exceptional performance is highly chain-length dependent. The shortest chain iPEG_9k is extremely brittle, fracturing at <1% strain with negligible toughness, and iPEG_21k remains brittle, breaking at only ∼13% strain with low energy absorption (*U*_T_ ≈ 0.9 MJ/m^3^). In contrast, once *M*_n_ reaches 41 kDa, the mechanical response changes qualitatively: *E* increases only slightly, but both ε_b_ and *U*_T_ increase by more than 2 orders of magnitude relative to iPEG_21k. This sharp transition from brittle (9 kDa, 21 kDa) to ultraductile (41 kDa) behavior at fixed ionic spacing, indicates that exceeding a critical chain length is necessary to build a continuous load-bearing network (discussed in detail in Sections 2.3 and 2.4). However, increasing chain length alone is insufficient to ensure ductility. Based on the same sample preparation and testing conditions, neat HO–PEG–OH_100k where chains are well-entangled in the melt state fails at <5% elongation.^46,50^

These macroscopic trends motivate our working hypothesis that introducing well-defined imidazolium centers into the PEG backbone can couple amorphous-phase reinforcement with altered semicrystalline reconfiguration during deformation (Figure 2). More specifically, we hypothesize that the high ductility arises from the following two factors in the amorphous and crystalline regimes, respectively.

(1) Ionic associations in the amorphous regime are sufficiently strong to act as transient physical cross-links,^69^ maintaining cohesion on deformation-relevant time scales; yet remain sufficiently dynamic to dissociate and reform under load (stick–slip hopping).^70–72^ Such dynamic constraints are expected to promote energy dissipation and contribute to strain hardening at large deformations.^3,73–76^

(2) Ionic modification suppresses lamellar thickening, yielding thinner crystallites without significantly increasing the long period. Sufficiently long chains (e.g., iPEG_41k) can effectively bridge adjacent crystallites,^76^ thereby increasing intercrystallite connectivity. Moreover, the crystal-mobile iPEG enables stress-induced crystalline destabilization and reorganization/recrystallization rather than brittle cracking.^77^

**Figure 2:**
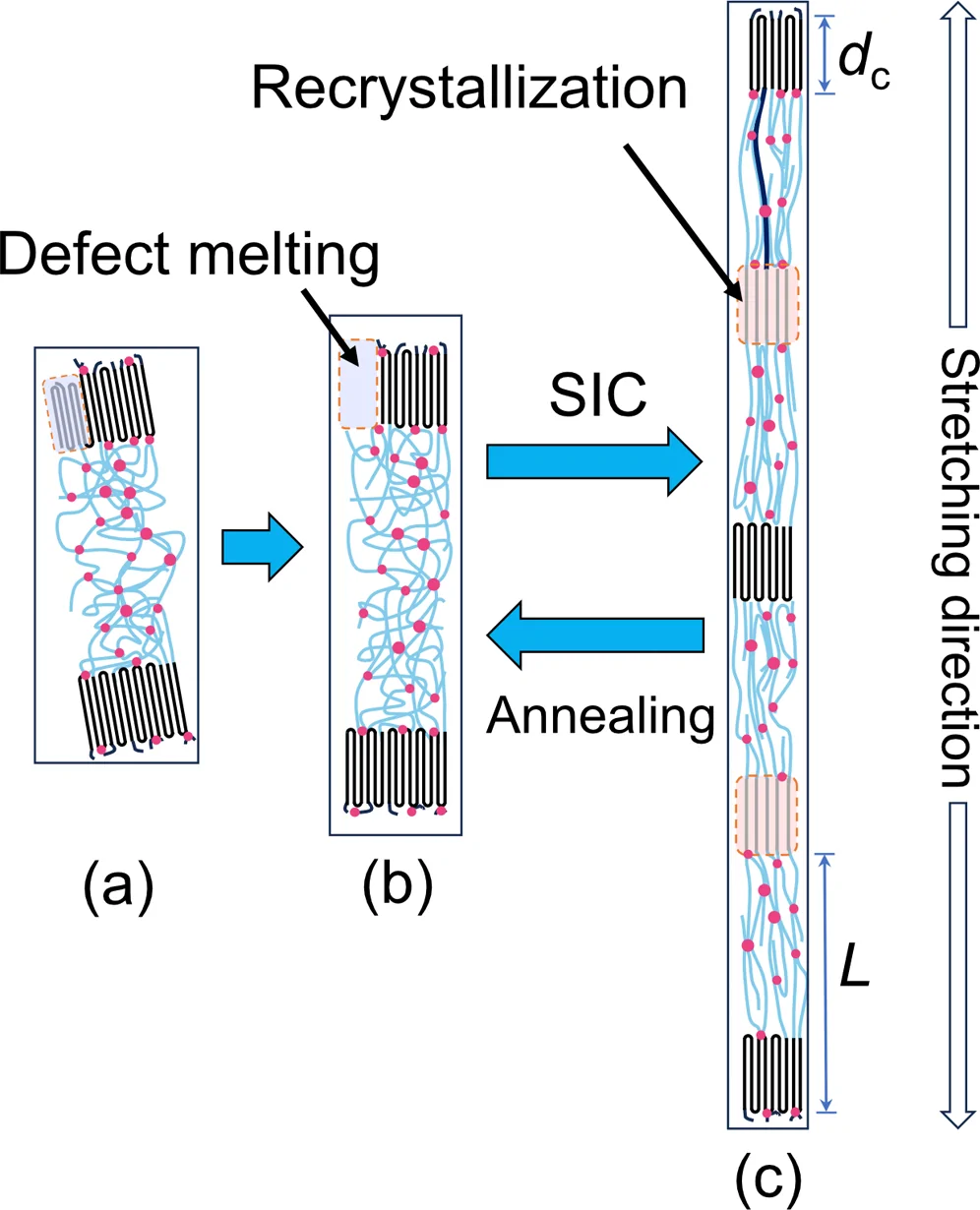
Proposed ion-mediated mechanism for the ultraductility of iPEG. (a) Initial semicrystalline state with randomly oriented crystallites connected through ionic associations in the amorphous phase. (b) Upon stretching, crystal defects undergo localized melting, enabling chain rearrangement and extension. (c) At larger strains, strain-induced crystallization (SIC) generates highly oriented and unfolded crystallites between neighboring crystalline domains. This adaptive melting-recrystallization process enables ultraductility and strain hardening. Because the newly formed crystallites are thermodynamically less stable than the original crystals, annealing at temperatures below the primary melting temperature can erase the oriented structures and induce shape recovery. The red dots indicate ionic groups while the highlighted chains represent tie chains.

In the following sections, we combine melt rheology to probe amorphous chain dynamics (Section 2.3) with SAXS/WAXS to quantify crystalline morphology and evolution during deformation (Section 2.4), with the goal of testing the role of ionic interactions in altering the mechanical response.

### 2.3 Melt Rheology Reveals Sticky-Ion Dynamics in the Amorphous Phase

The excellent toughness, stable necking, and pronounced strain hardening of iPEG suggest that the amorphous phase is not a passive interlamellar “soft layer”, but a load-bearing network in which long tie chains and entanglements continue to transmit stress at large deformation. To directly probe how ionic associations modify these amorphous-phase dynamics, we characterized the polymers in the melt (fully amorphous) at 60 °C, using oscillatory and rotational shear rheology (Figure 3). The zero-shear viscosity (η_0_), extracted from the low-shear rate plateau, increased sharply with *M*_n_. A power-law fit yielded η_0_ ∝ *M*_n_^4.3^, steeper than the η_0_ ∝ *M*_n_^3.2^ scaling reported for neutral PEO melts at the same temperature,^78^ consistent with long-lived, transient constraints arising from Im^+^/Br^–^ associations that add drag beyond ordinary entanglements. Similar behavior has been reported for strongly associating polyethylene-based polymeric ionic liquids (η_0_ ∝ *M*_v_^4.2^), and were attributed to electrostatic interactions between charged backbones and counterions.^79,80^

**Figure 3:**
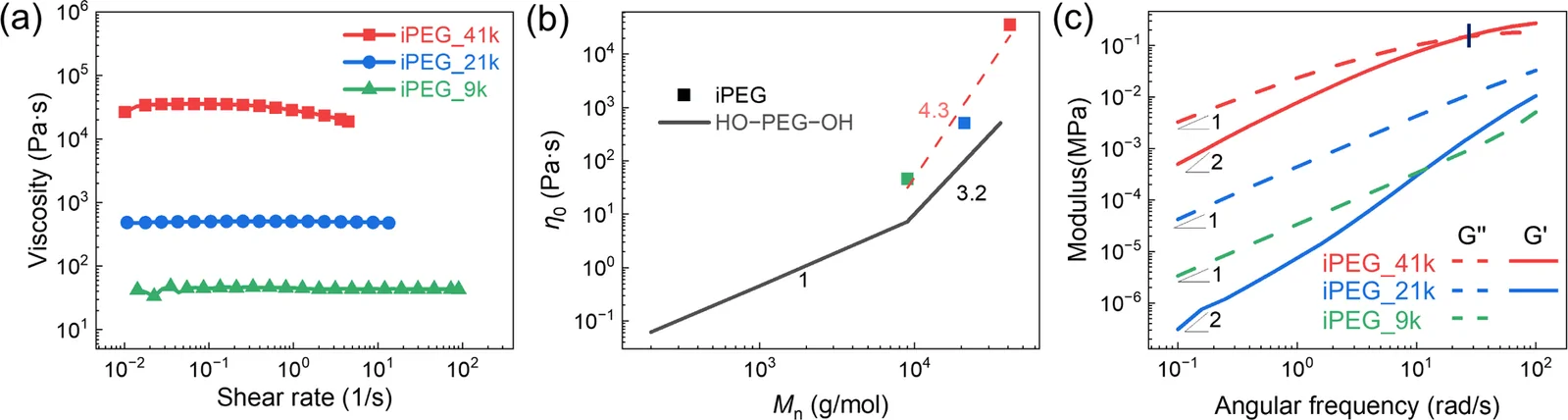
Rheology measurements for iPEGs at *T* = 60 °C reveal sticky-ion dynamics: (a) steady shear viscosity; (b) effect of *M*_n_ on steady shear viscosity of iPEG compared with HO–PEG–OH^78^ show enhanced viscosity scaling; and (c) frequency sweep curves indicate a long-lived ionic relaxation time. The HO–PEG–OH data in panel (b) were digitized and replotted from ref 78 with permission from Elsevier. Copyright 2012 Elsevier.

The frequency-dependent moduli reveal the molecular-weight dependence of this dynamic network, directly underpinning the mechanical threshold observed in Section 2.2. At 60 °C, iPEG_9k and iPEG_21k exhibited terminal flow behavior with the loss modulus exceeding the storage modulus (*G*″ > *G*′), whereas iPEG_41k exhibits a crossover of *G*′ and *G*″ at ω_c_ ≈ 25.5 rad·s^–1^, corresponding to a characteristic relaxation time τ_c_ = 1/ω_c_ ≈ 0.25 s, beyond which the whole chain relaxes (Figure 3c). Note that τ_c_ is determined by both net ionic association and molecular weight.^33^ Direct quantification of ion-pair lifetimes would require broadband dielectric spectroscopy or related kinetic measurements. Consistent with this more persistent associative response, a temperature-ramp measurement in torsional mode further shows that iPEG_41k retains a measurable *G*′ above its melting temperature, while the *G*′ of iPEG_21k decreases sharply upon melting and approaches a liquid-like response (Figure S7). Together, the crossover in iPEG_41k (but not in iPEG_9k/21k) and a measurable *G*′ above *T*_m_ indicate a molecular-weight threshold for sustaining associative connectivity on deformation-relevant time scales.

We therefore interpret that these dynamic physical cross-links reinforce the amorphous PEG matrix and facilitate load transfer between crystallites. Ionic interactions create transient connections between tails and unentangled loops from different crystallites, enhancing stress transmission and contributing to the material’s elastic modulus.

### 2.4 Crystallization Behavior and Deformation Mechanism

The remarkable mechanical behavior of iPEGs is further attributed to sufficient load-transfer connectivity and the ability of crystallites to reorganize under deformation. The former is enabled by thin lamellae (*d*_c_) and a moderate long period (*L*), while the latter requires high chain mobility and rapid nucleation and growth, both achieved in the iPEG design. To test these hypotheses, we quantitatively characterize the semicrystalline morphology of iPEGs and its evolution during mechanical loading.

#### 2.4.1 Ionic Modification Suppresses Lamellar Thickening while Preserving Intercrystallite Connectivity

Introducing imidazolium centers suppresses PEG crystallinity (Table 3). Across our iPEG series, *X*_c_ decreases from ∼61% to ∼30% (iPEG_9k to iPEG_41k), while ε_b_ increases from ∼0.9% to ∼2000%, a three-orders-of-magnitude change. Reported prior within-system studies on semicrystalline polypropylene and poly­(ethylene terephthalate) suggest that comparable reductions in crystallinity lead to much smaller gains in ε_b_ (often within 1–2 orders of magnitude).^81,82^ Thus, we speculate that the magnitude of ductility enhancement cannot be explained solely by *X*_c_ reduction. A control experiment supports this point (Figure 5): adding LiTFSI salt to HO-PEG–OH_100k ([Li]/[EO] = 0.06) reduces *X*_c_ from ∼70% to ∼17%, yet ε_b_ increases only from 4.4% to 26%. Moreover, salt-doped PEG demonstrates a two-orders-of-magnitude decrease in elastic modulus, while iPEG_41k maintains a comparable modulus to the neat PEG even at low *X*_c_. The salt-doped PEG also serves as a physical-blend control, showing that mobile, noncovalently attached ions can suppress crystallinity but cannot generate the persistent stress-transmitting junctions enabled by chain-bound ionic groups. This demonstrates that while lower crystallinity is beneficial for ultraductility, it is not the sole determinant.

**Table 3:** Crystallinity and Lamellar Dimensions of iPEGs and HO–PEG–OH

sample ID	*X*_c_ (%)	*T*_m_ (°C)	*L* (nm)^a^	*d*_c_ (nm)^b^	*d*_a_ (nm)^c^
iPEG_41k	30	43.3	15.0	4.5	10.5
iPEG_41k_d	39	43.5	12.3	4.8	7.5
iPEG_41k_q	30	42.8	12.4	3.7	8.7
iPEG _21k	49	44.1	13.3	6.5	6.8
iPEG _9k	61	46.6	11.6	7.1	4.5
HO–PEG–OH_100k	70	60.4	17.1	12.0	5.1
HO–PEG–OH_100k/salt	17	44.6	22.4	3.8	18.6

a Long period (*L*) was estimated based on SAXS.

b The crystal lamellar thickness (*d*_c_) was determined by *d*_c_ = *L*·*X*_c_.^37,45,92^ Note that *d*_c_ can be underestimated when *X*_c_ is low, where crystallites are dispersed in the amorphous matrix and *X*_c_ does not represent the local fraction of crystalline phase in each long period.

c The amorphous thickness (*d*_a_) was determined by *d*_a_ = *L* – *d*_c_.^9^

The sparse-ion character of iPEG_41k is also important. SAXS/WAXS measurements on molten iPEG_41k show no detectable additional scattering peak assignable to ionic aggregation (Figure S11, Supporting Information), consistent with the absence of pronounced nanoscale ionic cluster formation. This interpretation is supported by prior studies showing favorable ion-dipole and hydrogen-bonding interactions between PEG and imidazolium-based ionic species,^83–87^ although weakly correlated ionic clusters below the SAXS detection limit cannot be excluded. This sparse-ion regime is distinct from PEG-based ionenes with short ionic spacing (e.g., <1000 g/mol PEG per ion), where much higher ionic contents can fundamentally alter ion solvation and promote the formation of ionic-rich morphologies.^53,88^

The key lies in how ionic modification alters the lamellar dimensions. Compared to pristine PEG, ionic modification in iPEG_41 k leads to progressively thinner *d*_c_ (from 12.0 to 4.5 nm), while *L* also shows a moderate decrease (from 17.1 to 15 nm) (Figure 4 and Table 3). Within the iPEG series, the progressive decrease in *d*_c_ with increasing *M*_n_ likely reflects the slightly higher ionic content, which further perturbs regular PEG chain folding and suppresses lamellar thickening.^44,89^ Such a small lamellar thickness favors intercrystallite connectivity by tie chains, leading to mechanical stability. An even smaller *d*_c_ can be achieved by quenching iPEG from its melt state into liquid nitrogen to minimize crystal growth and lamellar thickening. The quenched sample (named as iPEG_41k_q) shows similar *X*_c_ (30%) but a smaller *d*_c_ (decreased from 4.5 to 3.7 nm) compared with its air-cooled counterpart (Table 3). Mechanically, quenching modestly lowers stiffness (*E*: 230 → 160 MPa) due to slightly reduced *d*_c_, while further increasing ductility (ε_b_: 1950% → 2430%) and slightly increasing toughness (*U*_T_: 311 → 342 MJ/m^3^) (Figure 5b and Table 2). This behavior supports the view that a morphology with thinner, likely less-perfected crystallites separated by a moderately thick amorphous layer is beneficial for accommodating large plastic deformation.^44–46,90,91^

**Figure 4:**
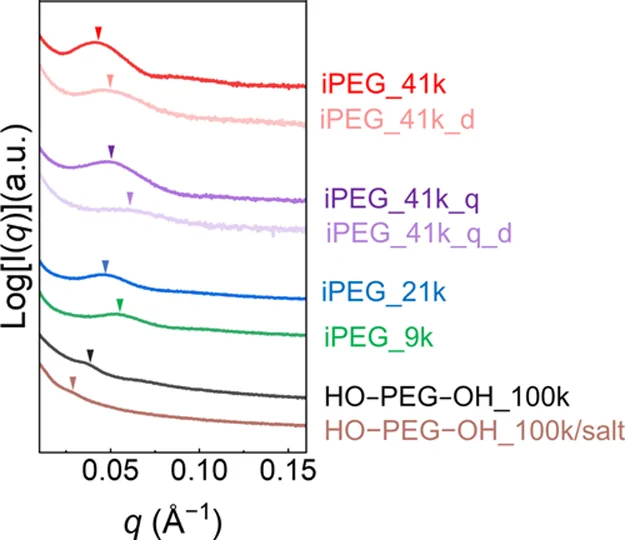
1D SAXS profiles of iPEGs and HO–PEG–OH, showing systematic shifts in the primary scattering maximum q* (and thus the long period *L* = 2π/q*).

**Figure 5:**
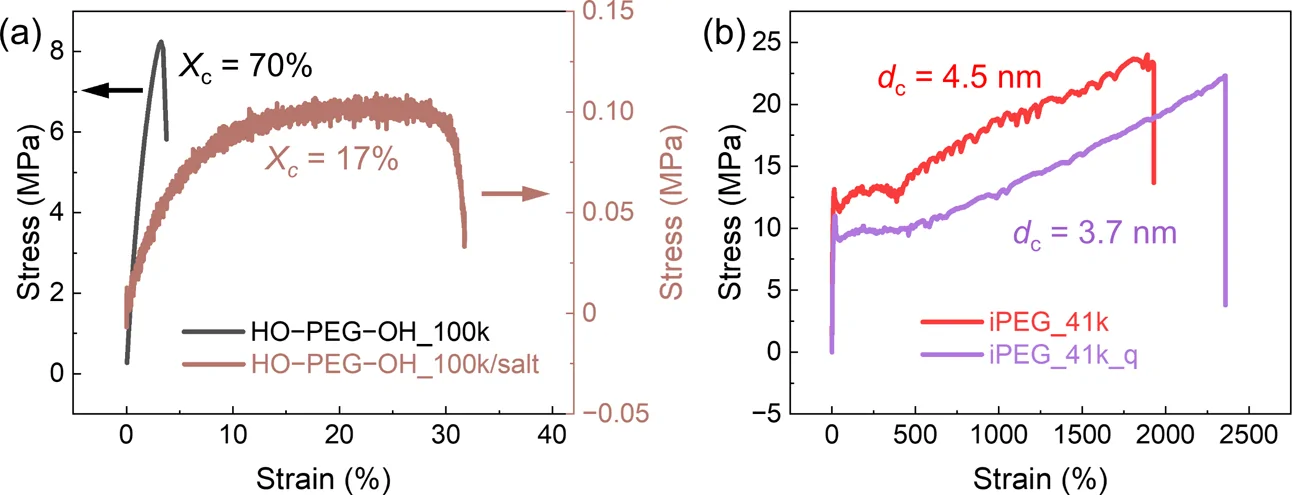
Control experiments distinguish the roles of crystallinity and lamellar morphology in ductility: (a) LiTFSI lowers *X*_c_ of HO–PEG–OH_100k to 17% but yields only modest extensibility; and (b) quenching iPEG_41k further reduces *d*_c_, increasing ductility.

To quantify intercrystallite connectivity among the iPEG series, we used the 1D Huang–Brown model^9,93–97^ to estimate the probability (*P*) that an amorphous chain emerging from a crystalline surface forms a tie chain bridging the adjacent crystallites (eq 1). We consider amorphous PEG to undergo random excursions between two parallel absorbing walls and only consider the projection of the chains in the stem direction (*z* direction). Thus, a tie chain is formed when the *z*-projection is greater than a critical distance, e.g., two lamellar thickness + amorphous layer thickness (2*d*_c_ + *d*_a_).

1 $$P=\frac{\int_{2d_{c}+d_{a}}^{\infty }d_{z}z^{2}\exp\left(-\frac{3z^{2}}{2Nb^{2}}\right)}{\int_{0}^{\infty }d_{z}z^{2}\exp\left(-\frac{3z^{2}}{2Nb^{2}}\right)}$$

where *N* is polymer’s degree of polymerization and *b* is the statistical segment length of PEG (see Supporting Information for details).

The *P* values for the iPEG series reveal a sharp molecular-weight dependence (Figure 6). While iPEG_9k shows negligible bridging probability, iPEG_21k has a moderate chance of forming bridges, and iPEG_41k exhibits a high probability of forming bridges (>30%). This emergence of a highly connected network of bridging chains in iPEG_41k is consistent with the observed mechanical and rheological thresholds for ultraductility. We emphasize that the Huang–Brown model evaluates only the geometric probability of intercrystallite bridging and does not describe ionic association strength or lifetime. Chain entanglements are a prerequisite for robust mechanical performance: effective stress transfer requires a connected amorphous network that bridges neighboring crystallites and capable of transmitting stress between crystalline domains. In iPEG_41k, the high tie-bridge probability provides this geometric connectivity, while ionic associations act as transient cross-links that constrain chain dynamics and thus increase the effective density of stress-transmitting junctions in the amorphous phase.

**Figure 6:**
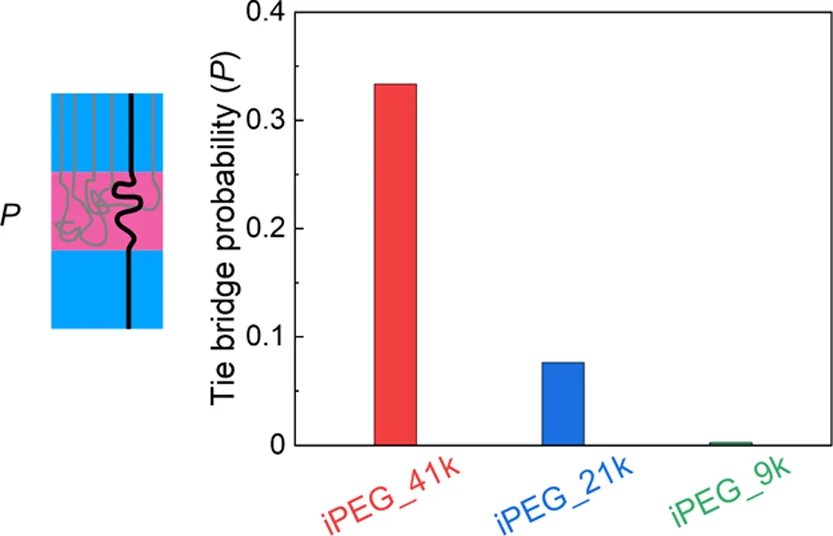
Tie-bridge probability (*P*) of iPEGs estimated using the 1D Huang–Brown model.

Note that in conventional semicrystalline polymers, *E* typically increases with *X*_c_ because the lamellar phase dominates stiffness.^1^ In iPEGs, however, *E* increases with *M*_n_ (iPEG_41k > iPEG_21k > iPEG_9k) when *X*_c_ decreases (Tables 2 and 3). This inversion indicates that *X*_c_ alone does not control the small-strain stiffness. Recent work has shown that the amorphous phase confined between lamellae can exhibit a modulus 1–2 orders of magnitude higher than bulk amorphous melt, owing to a higher density of stress-transmitting chains (tie molecules and entanglements) in the amorphous regions.^98^ By analogy, we propose that the higher-*M*_n_ iPEGs (with a slightly higher charge density) possesses a more constrained interlamellar amorphous phase, reinforced by both chain bridging and ionic associations. The resulting amorphous phase stiffening due to ionic cross-links offsets the modest reduction in *X*_c_. This interpretation is also consistent with recent “glassy ionogel” studies that dense, reversible ionic interactions can yield thermoplastic-like stiffness (hundreds of MPa up to ∼1 GPa) even in fully amorphous polymer networks.^27^

#### 2.4.2 Deformation-Induced Reorganization and Mechanistic Implications

Sections 2.3 and 2.4.1 establish the equilibrium structure and dynamics of iPEGs. However, the exceptional mechanical performance ultimately emerges under large deformation where the material is driven far from equilibrium.^4^ Ultraductility in semicrystalline polymers is often associated with the ability of crystalline domains to reorganize under applied stress rather than fracture catastrophically, e.g., strain-induced crystallization (SIC). As mentioned by Men and co-workers, during SIC, the stress is so high that the crystalline blocks are no longer stable and the deformation mechanism changes from block slips into a disaggregation-recrystallization process.^75^ To clarify how the semicrystalline structure of iPEG_41k evolves upon tensile deformation, 2D SAXS and WAXS patterns were collected before and after large-strain stretching, probing the nanoscale lamellar stacking and the crystalline lattice order,^99^ respectively. The undeformed iPEG_41k shows nearly isotropic SAXS and WAXS rings, reflecting randomly oriented PEG lamellae and crystallites (Figure 7a). Upon stretching to large strain, the WAXS pattern develops distinct equatorial crystalline arcs, while the SAXS signal becomes strongly anisotropic with meridional, streak-like scattering along the draw direction (Figure 7b). Quantitatively, stretching increases the overall crystallinity by roughly one-third (from 30 to 39%), while *d*_c_ changes only modestly (4.5 to 4.8 nm; Table 3). This trend is consistent with deformation-induced formation/reorganization of additional thin, oriented crystallites rather than thickening existing lamellae,^18,28–30,99,100^ as illustrated in Figure 2.

**Figure 7:**
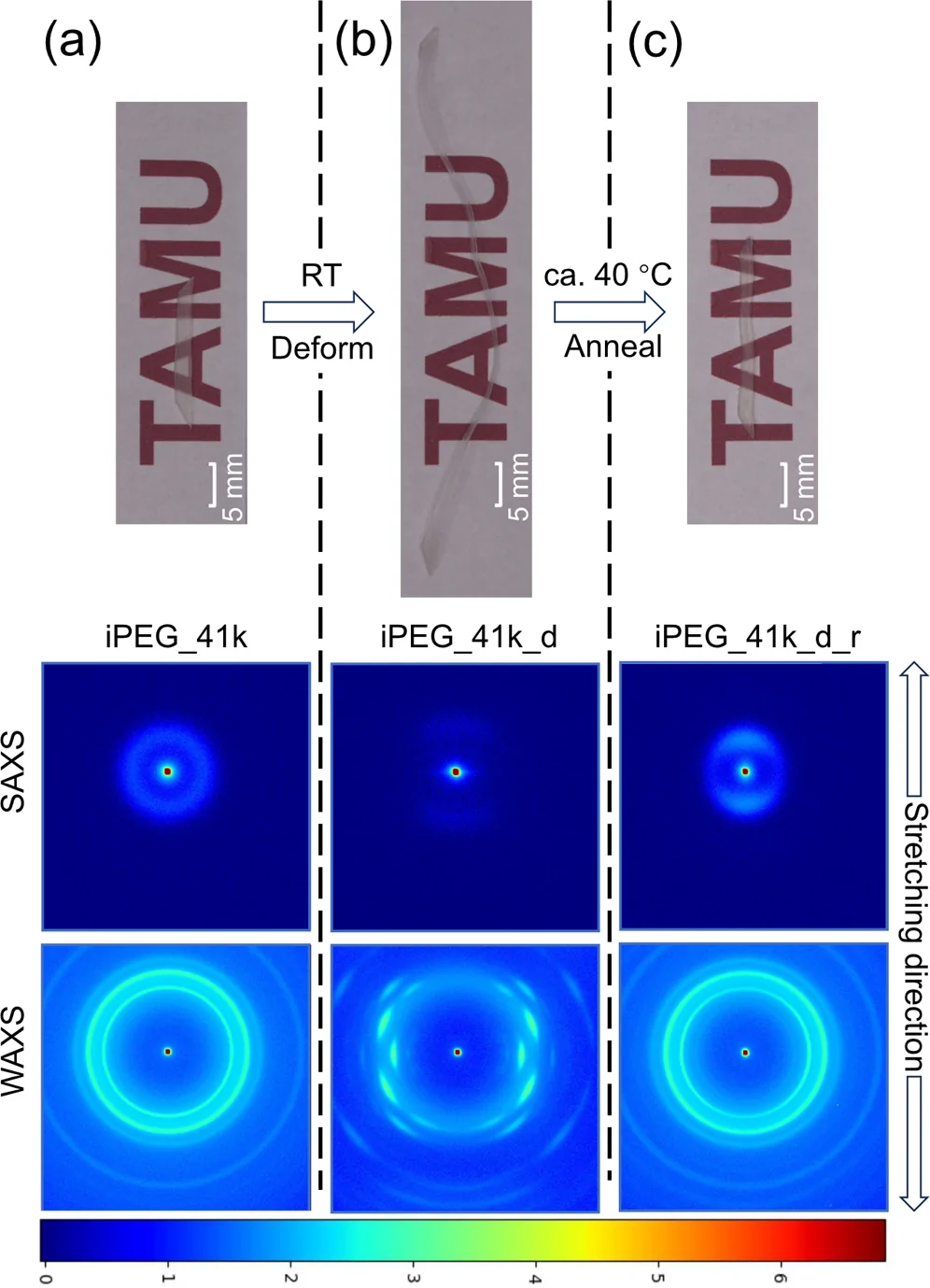
Macroscopic appearance and corresponding 2D SAXS/WAXS patterns of iPEG_41k (a) before stretching, (b) after stretching, and (c) after annealing at ca. 40 °C. Stretching transforms the initially isotropic SAXS and WAXS rings into highly anisotropic patterns, indicating the formation of oriented lamellar and crystalline structures along the stretching direction. Upon annealing, the sample contracts toward its original dimensions; crystalline orientation largely relaxes, although residual lamellar anisotropy remains. The reversible structural evolution demonstrates thermally induced recovery of the nonequilibrium oriented state.

Such thin lamellae formed during deformation are critical in preventing fracture, and we thus speculate that when iPEG chains are sufficiently long (41 kDa), the amorphous tie chains can dissipate energy without undergoing complete flow (as opposed to the shorter ones where the whole chain may have had relaxed), allowing new crystal nucleation and growth. Such dynamic crystal reorganization provides an effective mechanism for accommodating large plastic strains. To test our speculation, we annealed the deformed sample at around 40 °C, lower than the melting temperature of the primary crystal (ca. 43 °C) but likely higher than the newly formed thin crystallites. Such annealing releases the topological constraints of the SIC crystallites and allows relaxation of tie chains in the amorphous phase, leading to shape recovery to the original state where crystals are randomly oriented (Figure 7c).

Previous molecular simulation studies on semicrystalline HDPE also supports our speculation. They have shown that crystalline domains can undergo repeated destabilization (defect melting) and recrystallization during deformation at sufficiently slow strain rates.^101^ This behavior arises because slow deformation allows chain stems to redistribute between crystalline and amorphous regions, temporarily destabilizing crystallites and enabling their reorganization, which relieves local stresses and suppresses cavitation.

Notably, the stress–strain curves of iPEG_41k exhibit pronounced cyclic stress oscillations (drops and recoveries) in the plastic deformation regime, particularly at the slower nominal engineering strain rate (0.5 min^–1^) (Figure 8). Such oscillations have been reported in several semicrystalline polymers and are generally interpreted as a manifestation of intermittent microstructural rearrangement during deformation, although the underlying mechanism is not unique and may involve multiple necking, cavitation, thermal effects, or crystal destruction/reorganization depending on the material system.^102–109^ Local heating associated with deformation-induced melting/crystallization may contribute, but the fact that the oscillations are more pronounced at lower strain rate and have a long period (>1 min) makes temperature fluctuation unlikely to be the primary origin. In our case, the lamellae and crystal alignments observed in SAXS/WAXS, the postdeformation *X*_c_ increase, and the strain-rate dependence of the oscillations, are consistent with intermittent structural reorganization during plastic flow, potentially involving crystalline destabilization and recrystallization.^18,100,101^ However, time-resolved in situ SAXS/WAXS measurements will be required to establish a direct correlation between individual stress oscillations and crystallite destabilization/reorganization events. A detailed mechanistic study is warranted but beyond the scope of the present work.

**Figure 8:**
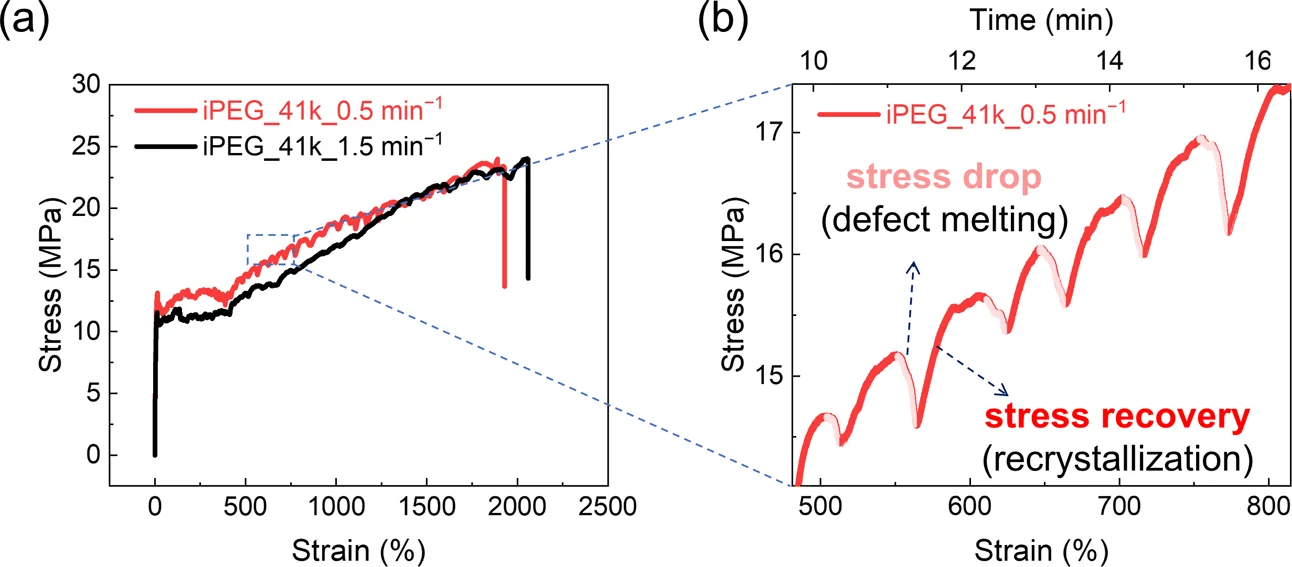
Cyclic stress serrations in iPEG_41k indicate the potential intermittent crystallite destabilization and recrystallization, leading to high ductility. Tests were performed at nominal engineering strain rates of 0.5 and 1.5 min^–1^ corresponding to crosshead speeds of 5 and 15 mm/min for an initial gauge length of 10 mm.

These observations point to a “time-window” requirement: intermittent crystal reorganization can contribute to stable plastic flow only if the amorphous phase maintains load-bearing connectivity long enough for such rearrangements to occur. Summarizing Sections 2.3 and 2.4, we attribute the ultraductility to a delicate balance between (i) connectivity (sufficient bridging chains and effective ionic tie points to maintain load transfer between crystallites long enough to enable reorganization); and (ii) reorganizability (ionic associations and crystallites that remain dynamic enough to reorganize under load).

This “connectivity-reorganizability” requirement also helps explain why introducing periodic ionic motifs into polyethylene (e.g., precise polyethylene ionomers/ionenes) does not necessarily improve room-temperature ductility relative to neat PE.^28,29,31^ In low-dielectric polyolefin matrices (nonpolar, less compatibility with ions), ionic motifs can form larger and more cohesive aggregates, like hard and quasi-permanent cross-links at room temperature, that kinetically hinder reorganization of ionic domains and crystallites during deformation.^30^ By contrast, polar PEG supports more dynamic ionic associations, enabling strain-induced reorganization rather than catastrophic fracture. It is also worth noting that the observed ultraductility relies on the intrinsic α_c_-mobility of PEG. In other words, although crystal-mobile polymers such as PEG possess the inherent ability to undergo large crystal plastic deformation, neat PEG remains brittle because it lacks an effective mechanism for stress transfer and energy dissipation between thick crystallites. The sparse and matrix-compatible ionic interactions reduce lamellar thickness and strengthen intercrystallite connectivity while preserving the intrinsic α_c_-mobility of PEG, thereby enabling extensive plastic deformation. Thus, α_c_-mobility should be regarded as a necessary, but not sufficient, prerequisite for this molecular design strategy.

Finally, benchmarking against industrial plastics further highlights the impact of this design. As summarized in Figure 9, iPEG_41k achieves a rare combination of modulus, strength, elongation at break, and toughness that matches or surpasses representative HDPE, isotactic polypropylene, and PEG-based polyurethane, while relying only on a simple linear PEG backbone with ∼1.5 mol % ionic groups.

**Figure 9:**
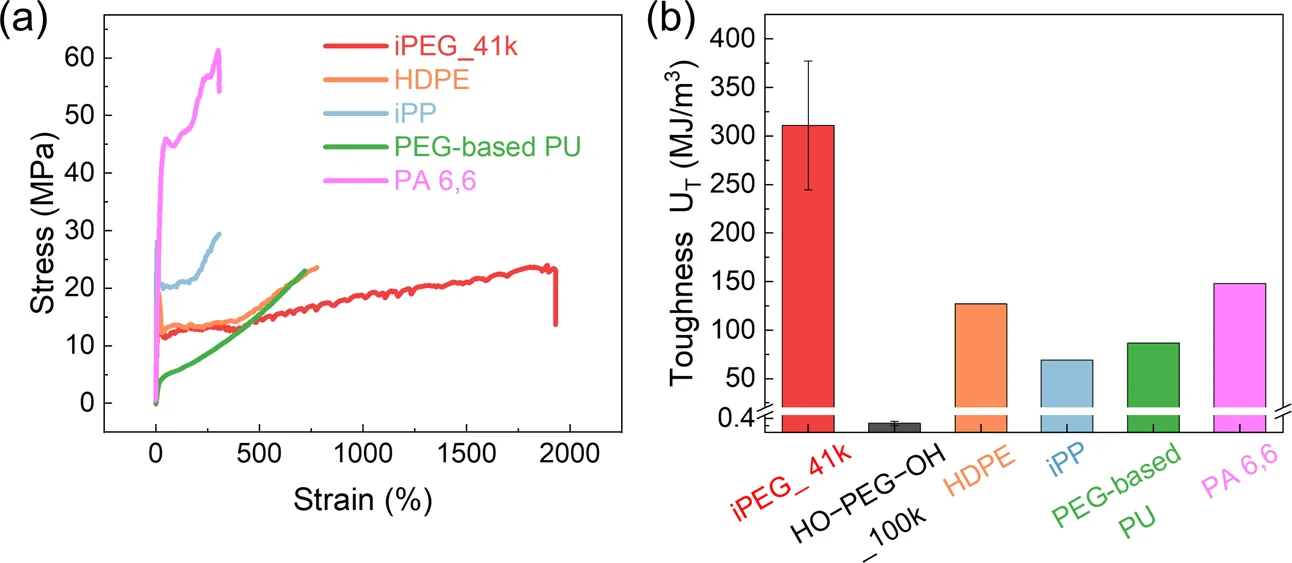
Benchmarking iPEG_41k against representative industrial plastic (high-density polyethylene,^11^ isotactic polypropylene,^11^ poly­(ethylene glycol)-based polyurethane^110^ and polyamide,^111^ literature data): iPEG_41k achieves an unusually high combination of strength, elongation, and toughness. Data for high-density polyethylene and isotactic polypropylene were digitized and replotted from ref 11 with permission from AAAS. Reproduced/modified from Eagan et al., *Science*, DOI: 10.1126/science.aah5744, 2017, AAAS. Data for poly­(ethylene glycol)-based polyurethane were digitized and replotted from ref 110, with permission from Elsevier. Copyright 1994 Elsevier. Polyamide data were reproduced/adapted from ref 111. Copyright 2024 American Chemical Society.

## 3 Conclusions

In this work, we demonstrate that sparse ionic functionalization can convert brittle semicrystalline PEG into an ultraductile and tough thermoplastic by sparsely introducing ionic motifs into a crystallizable backbone. Incorporating only ∼1.5 mol % imidazolium groups at fixed spacing (every 62 EO units) transforms PEG from a material that fails at a few percent strain into iPEG_41k, which combines high strength with ca. 2000% elongation and very high toughness, comparable to or exceeding representative industrial semicrystalline plastics. This unusual mechanical property profile arises from a coupled semicrystalline/ionic architecture: dynamic ionic associations generate long-lived transient constraints in the amorphous phase that sustain load-bearing connectivity, while ionic incorporation suppresses lamellar thickening to yield thin and reconfigurable PEG crystallites connected by effective tie chains that enable strain-induced reorganization rather than brittle fracture. Collectively, this coupled ionic/semicrystalline design provides a practical route to help overcome the typical stiffness-ductility trade-off in crystal-mobile semicrystalline polymers without introducing additional phases or permanent covalent cross-links. For our PEG-based materials specifically, the combination of a polar backbone, sparse ionic groups, melt processability and water processability may also be useful in applications involving polar interfaces or removable/water-processable materials.

More broadly, our results suggest a molecular design principle for toughening semicrystalline polymers with sparse ionic stickers: reinforce the amorphous phase while programming the semicrystalline morphology toward thin, deformation-reconfigurable lamellae. While the generality remains to be established experimentally, this strategy is most likely to translate to semicrystalline polymers that (i) possess sufficient intracrystalline mobility to permit crystal plasticity/reorganization, (ii) tolerate low levels of ionic functionalization without totally loss of crystallinity and (iii) maintain sufficient ion-backbone compatibility to avoid macrophase separation and allow reversible ionic associations. Exploring alternative ionic chemistries (counterion type, cation structure) and spacer length (ionic density), should open a versatile platform for engineering high-performance, recyclable plastics and structural electrolytes for emerging flexible and energy-relevant technologies.

## Supplementary Material







## Notes

Materials, synthetic procedures, Huang–Brown model, NMR spectra, tensile test, SAOS tests, DLS, DSC, TGA and SAXS/WAXS (PDF)
Supporting videos showing tensile deformation and thermal recovery of iPEG_41k (MP4)
Video S2 (MP4)

## Abbreviations

PEG: poly­(ethylene glycol)
iPEG: poly­(ethylene glycol) ionene
iPEG_41k: poly­(ethylene glycol) ionene with 41 kDa
iPEG_21k: poly­(ethylene glycol) ionene with 21 kDa
iPEG_9k: poly­(ethylene glycol) ionene with 9 kDa
iPEG_41k_q: iPEG_41k quenched into liquid nitrogen after hot-pressing
iPEG_41k_d: deformed iPEG_41k
*M*_n_: number-average molecular weight
*M*_v_: viscosity-average molecular weight
*N*: number of EO repeat units
*U*_T_: toughness
ε_b_: elongation at break
σ_b_: stress at break
*E*: Young’s modulus
η_0_: zero-shear viscosity
*G*″: loss modulus
*G*′: storage modulus
τ_c_: characteristic relaxation time
*X*_c_: crystallinity
*L*: long period
*d*_c_: crystal lamellar thickness
*d*_a_: amorphous thickness
