# Supplementary material for: From Brittle to Ultraductile: Precise Ionic PEG with Dynamic Networks and Reconfigurable Crystallites
Source: JACS Au. 2026 Aug 27;6(9):5275–86. doi: 10.1021/jacsau.6c00959 (PMC13625733; doi:10.1021/jacsau.6c00959)
Supplement: Supplementary file 1 [file au6c00959_si_001.pdf]

Supporting information for

From Brittle to Ultraductile: Precise Ionic PEG with Dynamic Networks and Reconfigurable Crystallites

Jie Xu,[1] Wenlin Zhang,[2] Hung-Jue Sue,[3] Shuyi Xie*[1]

[1] Department of Chemical Engineering, Texas A&M University, College Station, TX 77843, United States

[2] Department of Chemistry, Dartmouth College, Hanover, New Hampshire, NH 03755, United States

[3] Polymer Technology Consortia, Department of Materials Science and Engineering, Texas A&M University, College Station, TX, 77843, United States

E-mail: shuyixie@tamu.edu

Table of Contents

1. Experimental Section **S3**

1.1 Materials S3

1.2 Synthetic Procedures S3

1.2.1 Procedures for α,ω-imidazole PEG S3

1.2.2 Procedures for α,ω-bromo PEG S5

1.2.3 Procedures for iPEGs S5

1.2.4 Procedures for HO-PEG-OH_100k/salt S6

2. Material Characterization **S7**

2.1 Proton nuclear magnetic resonance (^{1}H NMR) spectroscopy S7

2.2 Dynamic light scattering (DLS) measurements S9

2.3 Tensile test S10

2.4 Small-amplitude oscillatory shear (SAOS) Tests S12

2.5 Differential scanning calorimetry (DSC) S13

2.6 Thermogravimetric analysis (TGA) S15

2.7 Small- and wide-angle X-ray scattering (SAXS/WAXS) S16

3. Huang-Brown Model **S19**

4. References **S20**

1. Experimental Section

1.1 Materials

All reagents were purchased from commercial suppliers and used without further purification unless stated otherwise. Polyethylene glycol (diol PEG) was dried under vacuum dry at 70 °C for several hours prior to use. Polyethylene glycol (diol PEG, M_n = 2800 g/mol) was obtained from Tokyo Chemical Industry, while polyethylene glycol (HO-PEG-OH_100k, M_v = 100,000 g/mol) was purchased from Sigma-Aldrich. Triethylamine (TEA, ≥ 99.5%), methanesulfonyl chloride (MsCl, ≥ 99.7%), sodium hydride (NaH, 60% dispersion in mineral oil), imidazole (≥ 99%), 6-bromohexanoyl chloride (97%) and lithium bis(trifluoromethanesulfonyl)imide (LiTFSI, anhydrous, ≥ 99.99% trace metals basis) were acquired from Sigma-Aldrich. Solvents, including dichloromethane (DCM, anhydrous, contains 40-150 ppm amylene as stabilizer, ≥ 99.8%), water (HPLC grade), tetrahydrofuran (THF, anhydrous, ≥ 99.9%), methanol (MeOH, anhydrous, ≥ 99.9%) and chloroform ($CHCl_3$, anhydrous, contains amylenes as stabilizer, ≥ 99%) were obtained from Sigma-Aldrich. Diethyl ether (anhydrous, ≥ 99.9%), ethanol (EtOH, anhydrous, ≥ 99.5%) and chloroform-d (≥99.8 atom %D) was obtained from Fisher Scientific. Ultra-high purity nitrogen was obtained from Airgas.

1.2 Synthetic Procedures

1.2.1 Procedures for α,ω-imidazole PEG

<u>Procedures for α,ω-mesylate PEG</u>

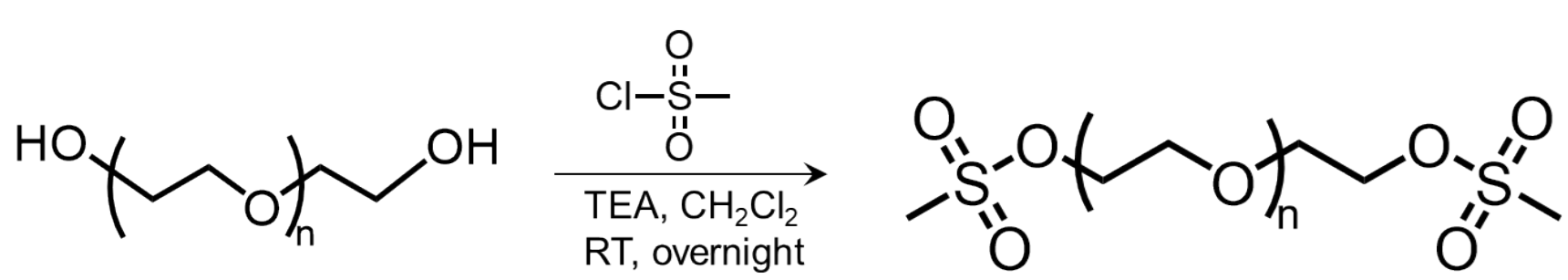

α,ω-imidazole PEG was prepared from a purified α,ω-diol PEG precursor (M_n = 2800 g/mol) via end-group activation under standard mesylation conditions. Briefly, diol PEG (3.0 g, 1 mmol) was dissolved in anhydrous DCM in a 100 mL round bottom flask, and triethylamine (0.80 g, 8 mmol) was added with stirring. After N_2 purging (30 min), the solution was cooled to 0 °C, and

methanesulfonyl chloride (0.92 g, 8 mmol) was introduced dropwise. The cooling bath was removed and the reaction was allowed to proceed at room temperature overnight. The mixture was then washed with water (30 mL); phases were separated by brief vortexing (1 min) and centrifugation (3500 rpm, 5 min), and the organic layer was collected. The polymer was isolated by precipitation into cold diethyl ether and dried under vacuum at room temperature for 48 hours to afford the α,ω-mesylated PEG as a white solid with near-quantitative end-group fidelity. ^{1}H-NMR (400 MHz, Chloroform-d, ppm): δ = 4.39−4.37 (t, 4H, CH_2), 3.83−3.45 (m, CH_2), 3.08 (s, 6H, CH_3).

<u>Procedures for α,ω-imidazole PEG</u>

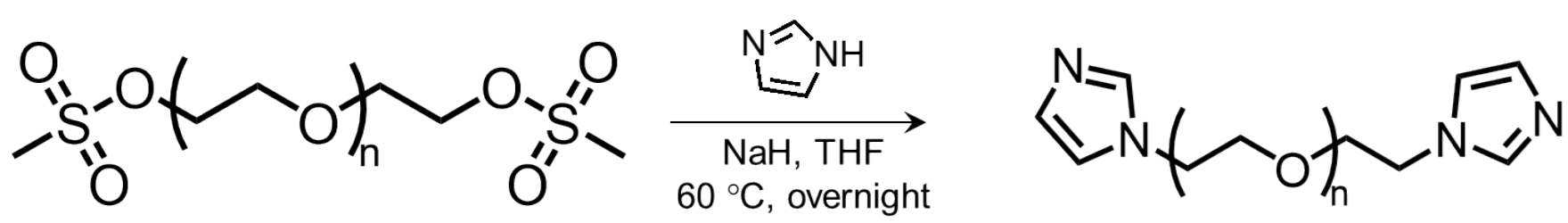

α,ω-mesylate PEG was prepared by nucleophilic substitution of the α,ω-mesylated PEG. Briefly, α,ω-mesylate PEG (0.67 mmol, from last step) was dissolved in anhydrous THF (8 mL) in a 25 mL round-bottom flask and degassed under N_2 for 30 min. Separately, NaH (0.064 g, 2.67 mmol) and imidazole (0.18 g, 2.67 mmol) were combined in a 100 mL Schlenk flask inside a glovebox. The flask was connected to a Schlenk line and anhydrous THF (4 mL) was added to generate the imidazolide (H_2 evolution vented through the line). The mesylate-PEG solution was then added dropwise to the stirred imidazolide suspension. The reaction mixture was stirred at 60 °C overnight (aluminum bead bath, Lab Armor). After cooling to room temperature, the reaction was quenched by dropwise addition of MeOH (1 mL). The polymer was isolated by precipitation into cold diethyl ether twice, and dried under vacuum at room temperature for 24 hours, followed by additional drying at 60 °C for 24 hours to afford α,ω-imidazole PEG as a white powder with near-quantitative end-group fidelity. ^{1}H-NMR (400 MHz, Chloroform-d, ppm): δ = 7.53 (s, 2H, CH), 7.04 (s, 2H, CH), 6.99 (s, 2H, CH), 4.12−4.10 (t, 4H, CH_2), 3.83−3.45 (m, CH_2).

1.2.2 Procedures for α,ω-bromo PEG

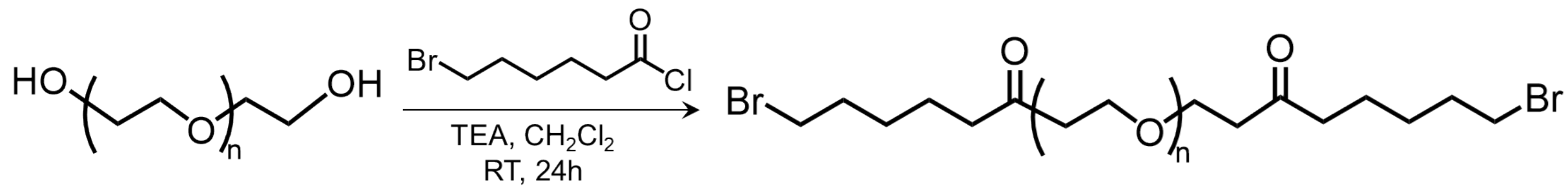

α,ω-bromo PEG was synthesized from a purified α,ω-diol PEG precursor (M_n = 2800 g/mol) by acylation with 6-bromohexanoyl chloride. Briefly, α,ω-diol PEG (10g, 3.6 mmol) and triethylamine were dissolved in anhydrous CH_2Cl_2 (40 mL) in a 250 mL round-bottom flask and degassed under N_2 for 30 min. In parallel, 6-bromohexanoyl chloride (1.7 g, 7.9 mmol) was dissolved in anhydrous CH_2Cl_2 (20 mL) and degassed under N_2 for 30 min. The 6-bromohexanoyl chloride solution was added dropwise to the PEG/TEA solution at 0 °C, and the mixture was stirred for 1 hour. The reaction was then allowed to warm gradually to room temperature and stirred for 24 hours. After completion, the solvent was removed under reduced pressure and the residue was further dried under vacuum at room temperature for 24 hours. The crude polymer was redissolved in MeOH (10 wt%) and purified by precipitation into diethyl ether to remove small-molecule impurities. The precipitated polymer was collected by filtration and dried under vacuum at room temperature for 24 hours, followed by additional drying at 60 °C for 24 hours to afford α,ω-bromo PEG as a white powder with near-quantitative end-group fidelity. ^{1}H-NMR (400 MHz, Chloroform-d, ppm): δ = 4.24−4.21 (t, 4H, CH_2), 3.83−3.45 (m, CH_2), 3.42−3.39 (t, 4H, CH_2), 2.37−2.33 (m, 4H, CH_2), 1.91−1.84 (m, 4H, CH_2), 1.68−1.62 (m, 4H, CH_2), 1.51−1.44 (m, 4H, CH_2).

1.2.3 Procedures for iPEGs

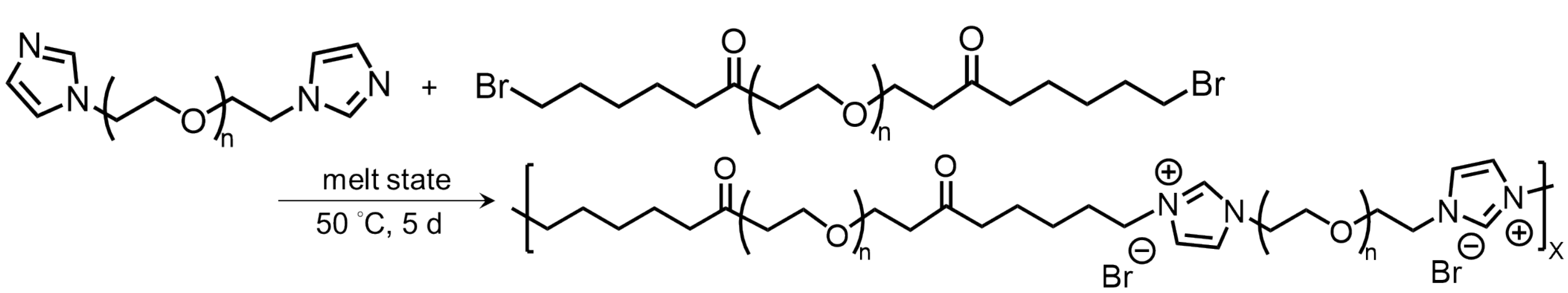

α,ω-imidazole PEG and α,ω-bromo PEG were combined in a 25 mL round-bottom flask. To obtain a series of linear iPEGs, the molar feed ratio of α,ω-imidazole PEG to α,ω- bromo PEG

was varied (1:1, 1:0.75, and 1:0.5). In a glovebox, the mixture was heated at 50 °C and stirred for 5−10 days in an aluminum bead bath under solvent-free conditions. This neat Menschutkin polyquaternization afforded linear iPEGs with targeted number-average molecular weights ($M_n \approx$ 9, 21, and 41 kDa) while maintaining a constant ionic spacing ($N = 62$). For iPEG_41k: ^{1}H-NMR (400 MHz, Chloroform-d, ppm): δ = 10.51 (s, CH), 7.68 (s, CH), 7.30 (s, CH), 4.64−4.61 (t, CH_2), 4.31−4.27 (t, CH_2), 4.22−4.20 (t, CH_2), 3.91−3.44 (m, CH_2), 2.37−2.33 (m, CH_2), 1.97−1.91 (m, CH_2), 1.71−1.62 (m, CH_2), 1.44−1.37 (m, CH_2). For iPEG_21k and iPEG_9k: ^{1}H-NMR (400 MHz, Chloroform-d, ppm): δ = 10.55 (s, CH), 7.68 (s, CH), 7.53 (s, 2H, CH), 7.30 (s, CH), 7.03 (s, 2H, CH), 6.99 (s, 2H, CH), 4.64−4.61 (t, CH_2), 4.31−4.27 (t, CH_2), 4.22−4.20 (t, CH_2), 4.12−4.10 (t, CH_2), 3.91−3.44 (m, CH_2), 2.38−2.34 (m, CH_2), 1.98−1.91 (m, CH_2), 1.72−1.64 (m, CH_2), 1.45−1.37 (m, CH_2).

1.2.4 Procedures for HO-PEG-OH_100k/salt

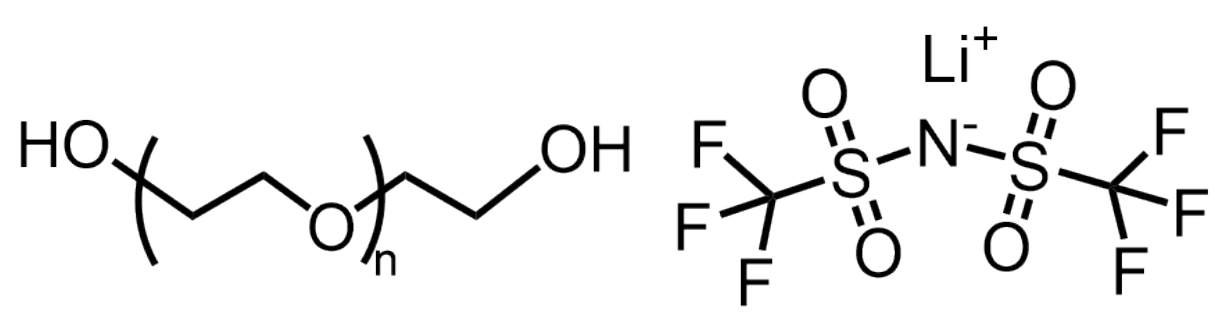

Purified α,ω-diol PEG (M_v = 100,000 g/mol; 0.30 g) and LiTFSI (0.117 g) were charged into a 25 mL round-bottom flask, followed by addition of MeOH (3 mL) and chloroform (2 mL). Inside a glovebox, the mixture was heated to 50 °C and stirred for 3 days in an aluminum bead bath. The solvents were then removed under reduced pressure, and the residue was dried under vacuum at room temperature for 24 h, followed by additional drying at 60 °C for 24 h.

2. Material Characterization

2.1 Proton nuclear magnetic resonance (^1H NMR) spectroscopy

^1H NMR spectroscopy was recorded on a Bruker Avance Neo 400Hz spectrometer. Chloroform-d (≥ 99.8 atom %D, anhydrous) was used as the solvent to ensure complete polymer dissolution and minimize signal overlap. Spectra were collected at 25 °C using 16 scans and a 15 s relaxation delay between scans.

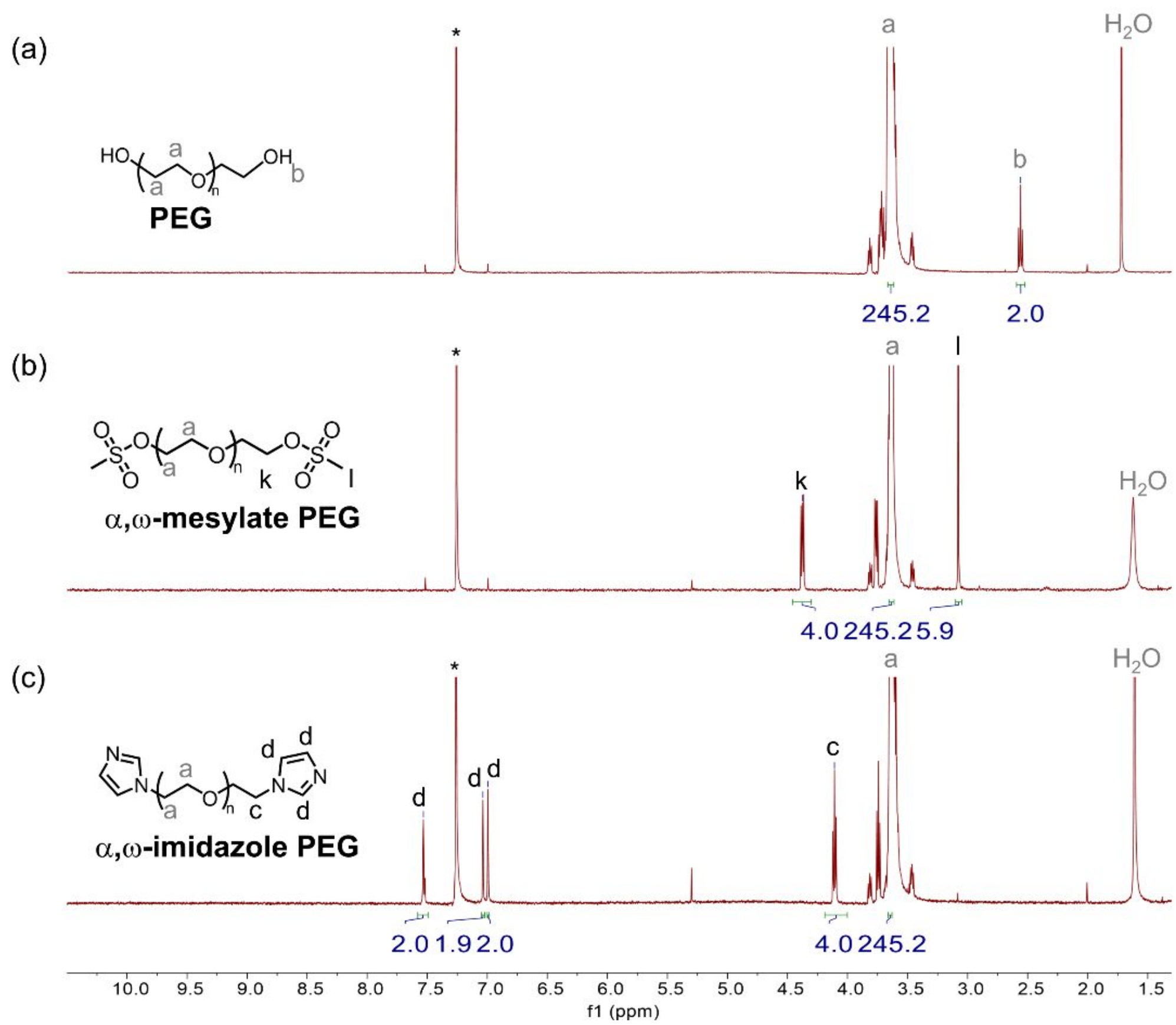

Figure S1. ^1H NMR spectra (in $CDCl_3$*) of (a) α,ω-diol PEG (M_n = 2800 g/mol), (b) α,ω-mesylate PEG and (c) α,ω-imidazole PEG.

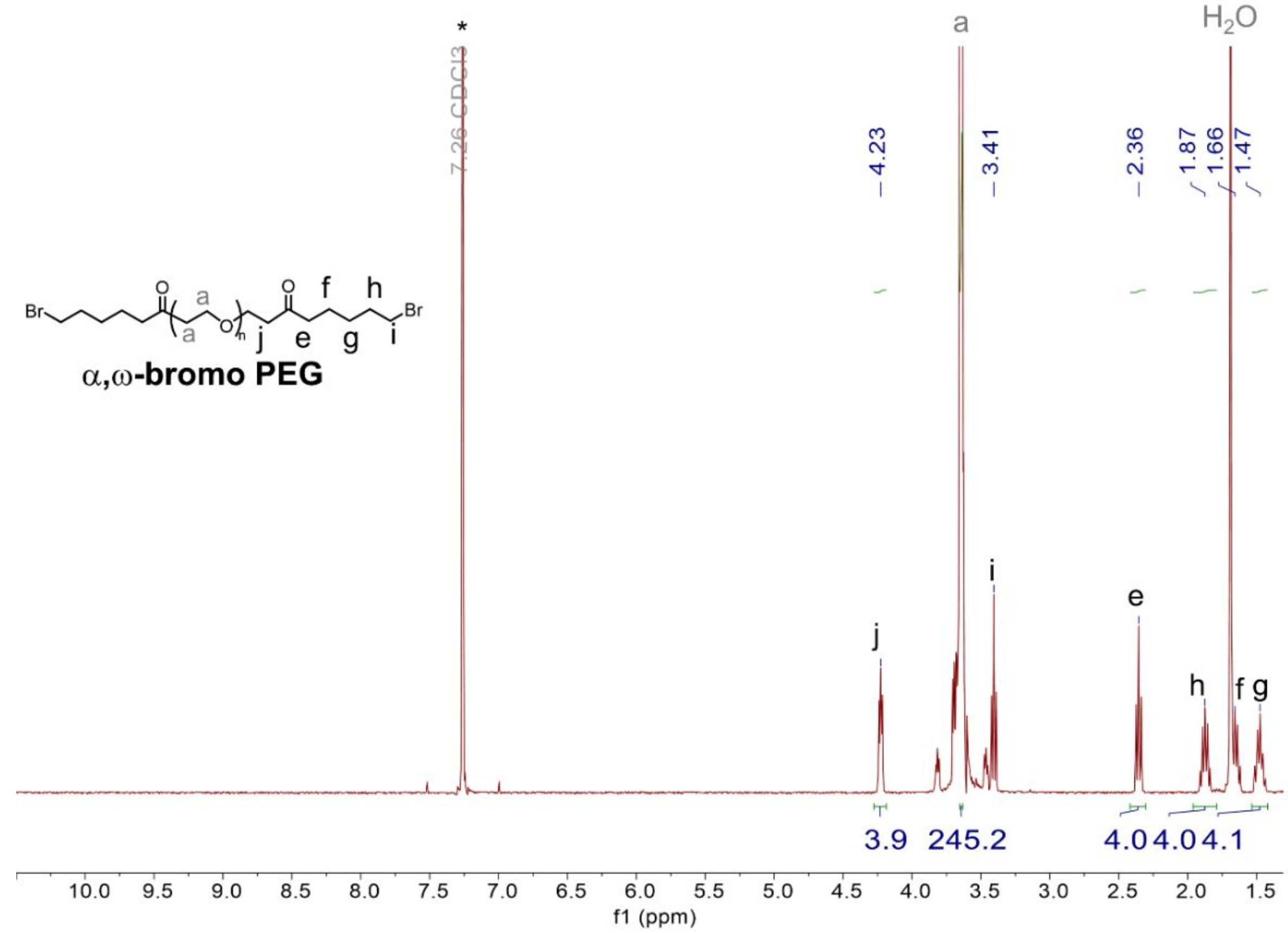

Figure S2. ^{1}H NMR spectrum (in $CDCl_3$*) of α,ω-bromo PEG.

134

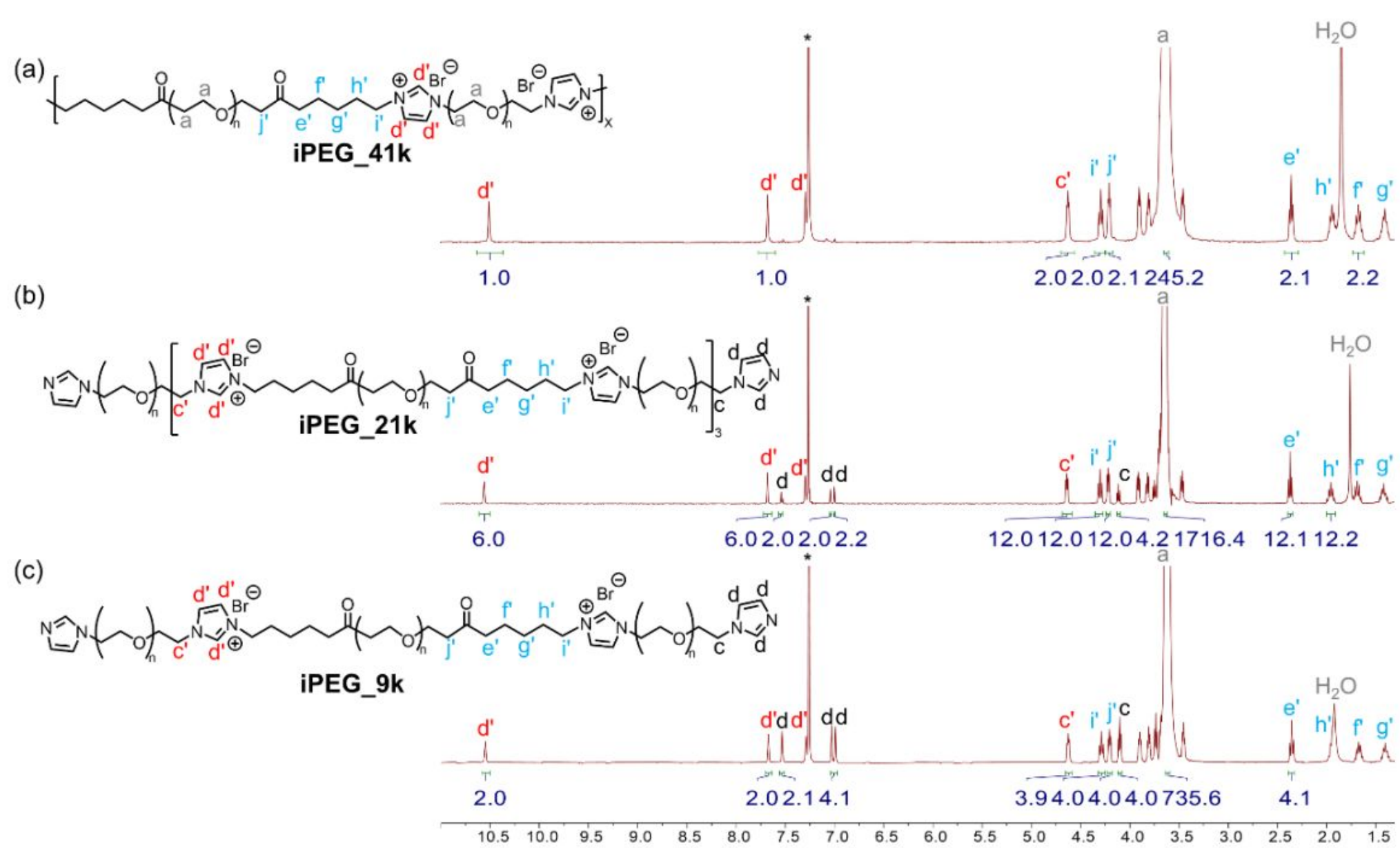

Figure S3. ^{1}H NMR spectra (in $CDCl_3$*) of (a) iPEG_41k, (b) iPEG_21k and (c) iPEG_9k.

2.2 Dynamic light scattering (DLS) measurements

Dynamic light scattering measurements of the iPEG/THF solutions were performed using a Malvern Zetasizer Nano ZS equipped with a laser light source and backscatter detection (173°). Prior to analysis, the solutions were filtered through a 220 nm syringe filter directly into a quartz cuvette. To ensure cleanliness, the cuvette was rigorously rinsed with pure THF before each experiment. Each sample was measured 5−6 times to determine the average hydrodynamic radius (R_h). The relationship between the hydrodynamic radius and molecular weight is described by equation (1)[1]:

$$R_h \propto R_g = \sqrt{\frac{Nb^2}{6}} \qquad (1)$$

where R_h and R_g are hydrodynamic radius and radius of gyration, respectively; b is the statistical segment length and N is the degree of polymerization.

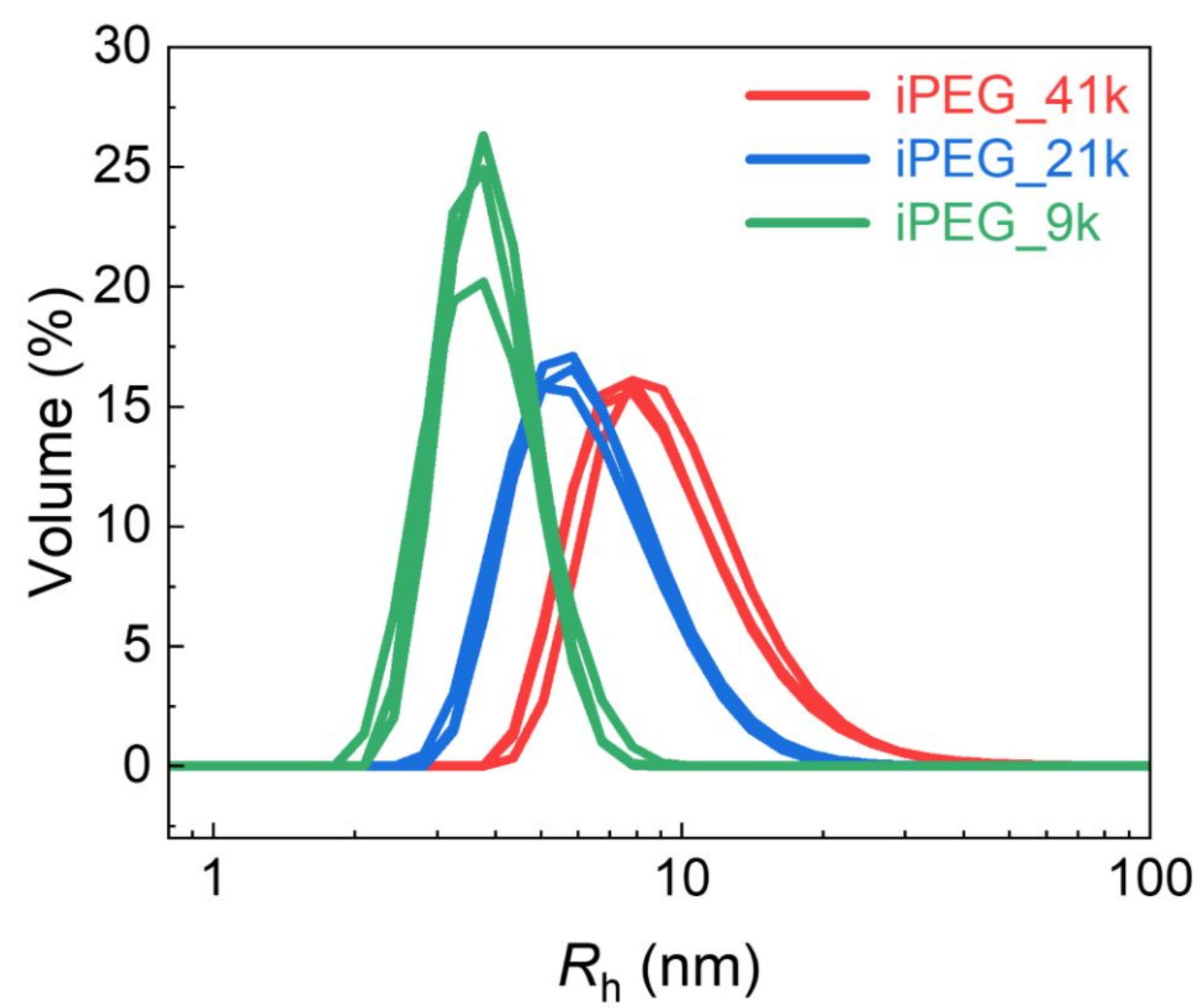

Figure S4. Dynamic light scattering (DLS) size distributions of iPEGs, showing average hydrodynamic radii (R_h) of 7.7 nm (iPEG_41k), 5.7 nm (iPEG_21k), and 3.6 nm (iPEG_9k).

2.3 Tensile tests

Tensile tests were performed on an Instron 6800 Universal Testing Machine equipped with a 500 N load cell using a nominal engineering strain rates of 0.5 min^{-1} or 1.5 min^{-1} until sample failure. The initial gauge length was around 10 mm, corresponding to crosshead speeds of 5 and 15 mm/min, respectively. Material toughness (U_T; MJ/m^3) was determined by integration of the tensile curve. Tensile statistics were reported according to the average and standard deviation of independent tensile tests. Five independent measurements were performed for iPEG_41k; other samples were measured using two to four specimens depending on sample availability. Specimens were prepared via hot pressing at 180 °F for 3 minutes. After removal, they were allowed to cool to room temperature and subsequently cut into tensile bars with dimensions of approximately 40 mm × 2 mm× 0.3mm (length × width × thickness).

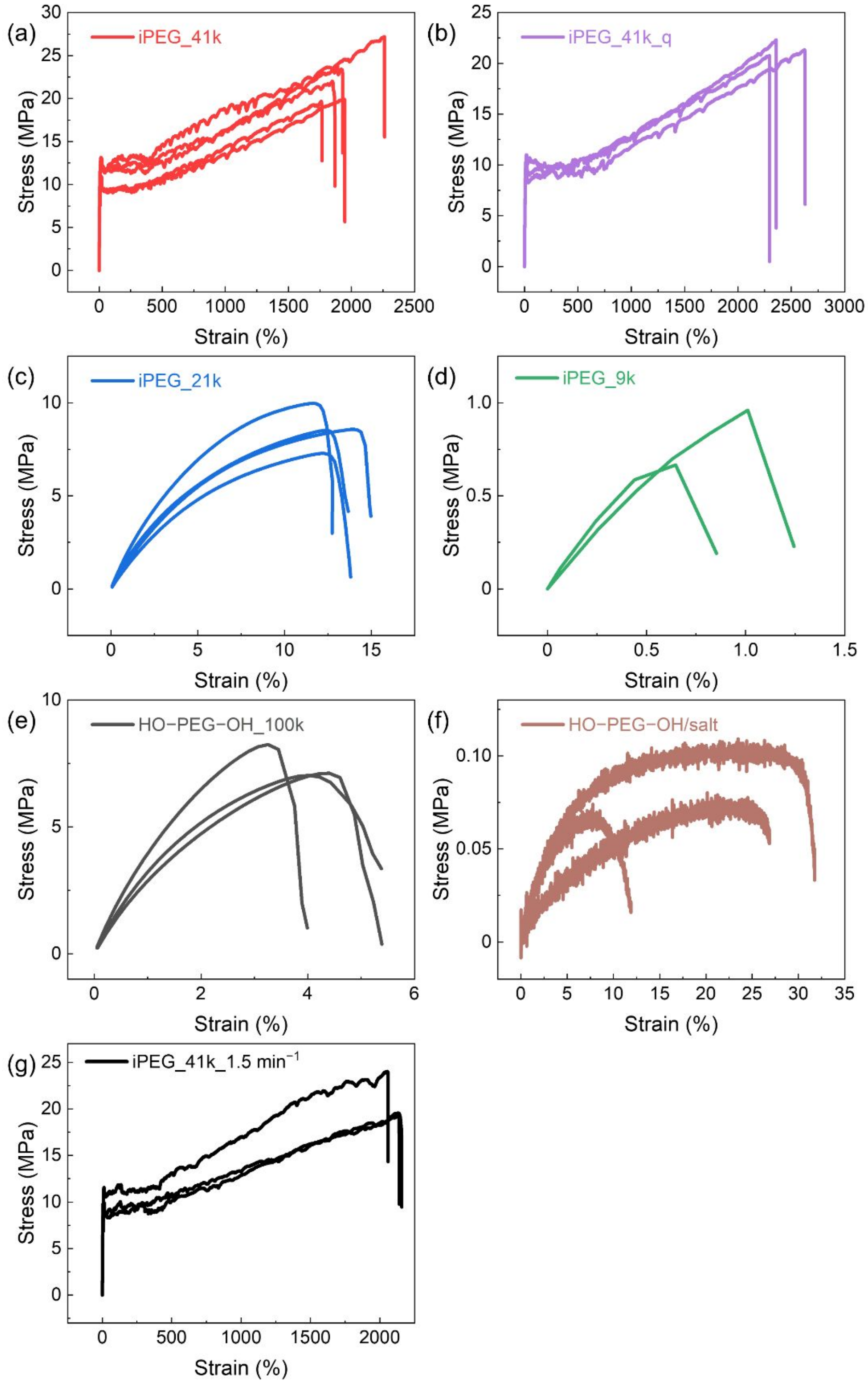

Figure S5. Tensile stress–strain curves of iPEGs and HO−PEG−OH measured at 0.5 min^{-1} (a−f); panel (g) shows iPEG_41k tested at 1.5 min^{-1}.

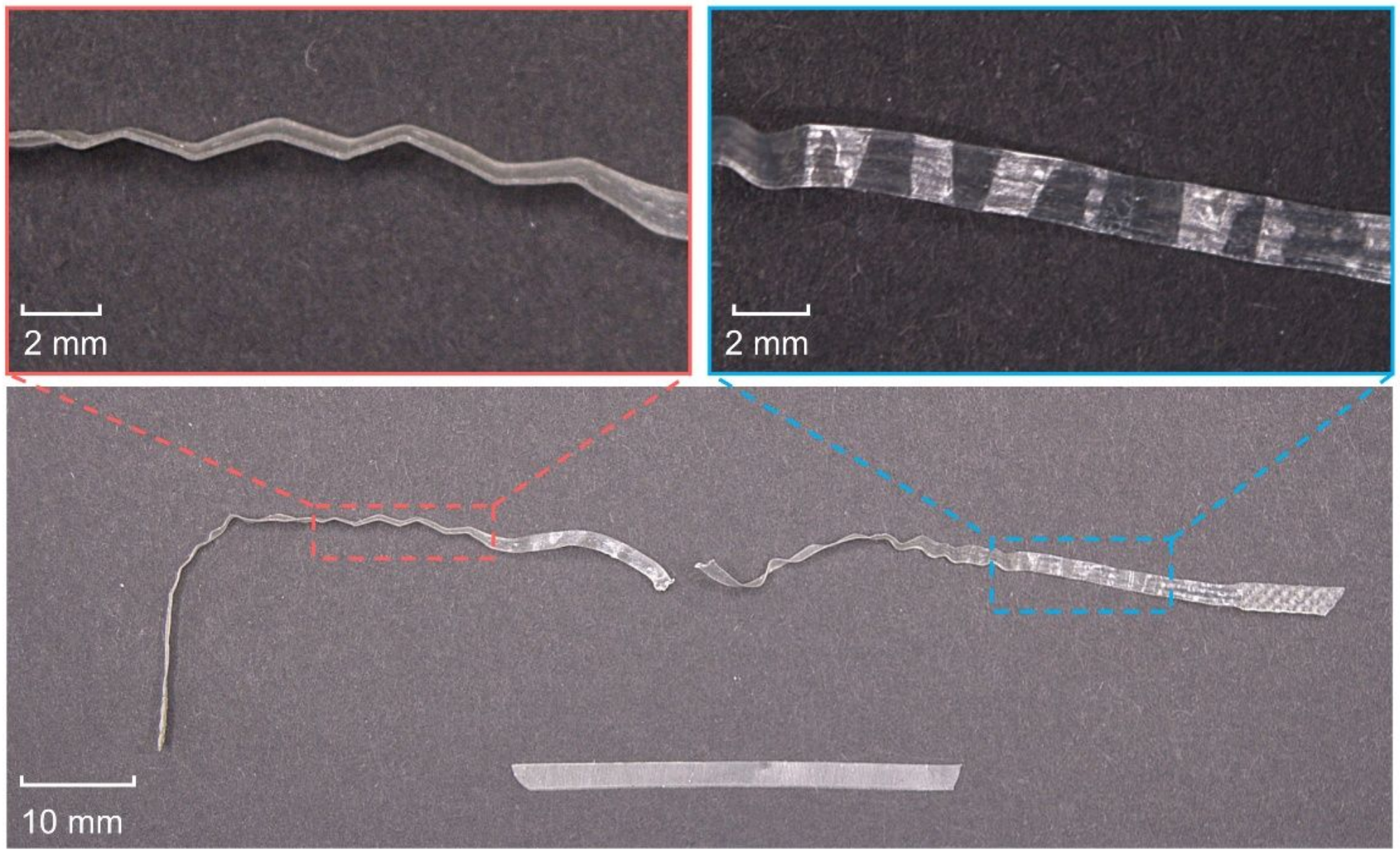

Figure S6. Photographs of iPEG_41k before and after tensile tests.

2.4 Small-amplitude oscillatory shear (SAOS) tests

Rheological measurements were conducted using a TA Instruments AR-G2 rheometer equipped with a 25 mm parallel-plate geometry. An N_2 inert gas purge was employed to prevent atmosphere exposure, and a liquid nitrogen (LN_2) cooling system facilitated temperature control. Steady shear experiments and dynamic shear experiments were performed at 60 °C for all the samples. The strain amplitudes used for dynamic shear measurements were confirmed to be within the linear viscoelastic (LVE) region. The angular frequency ranged from 0.1 rad/s to 100 rad/s.

A temperature-ramp experiment was performed on an ARES-G2 rheometer (TA Instruments) operated in torsional mode. Measurements were conducted at a strain amplitude of 0.05% and a fixed frequency of 1 Hz while the temperature was increased from 30 to 90 °C at 3 °C/min.

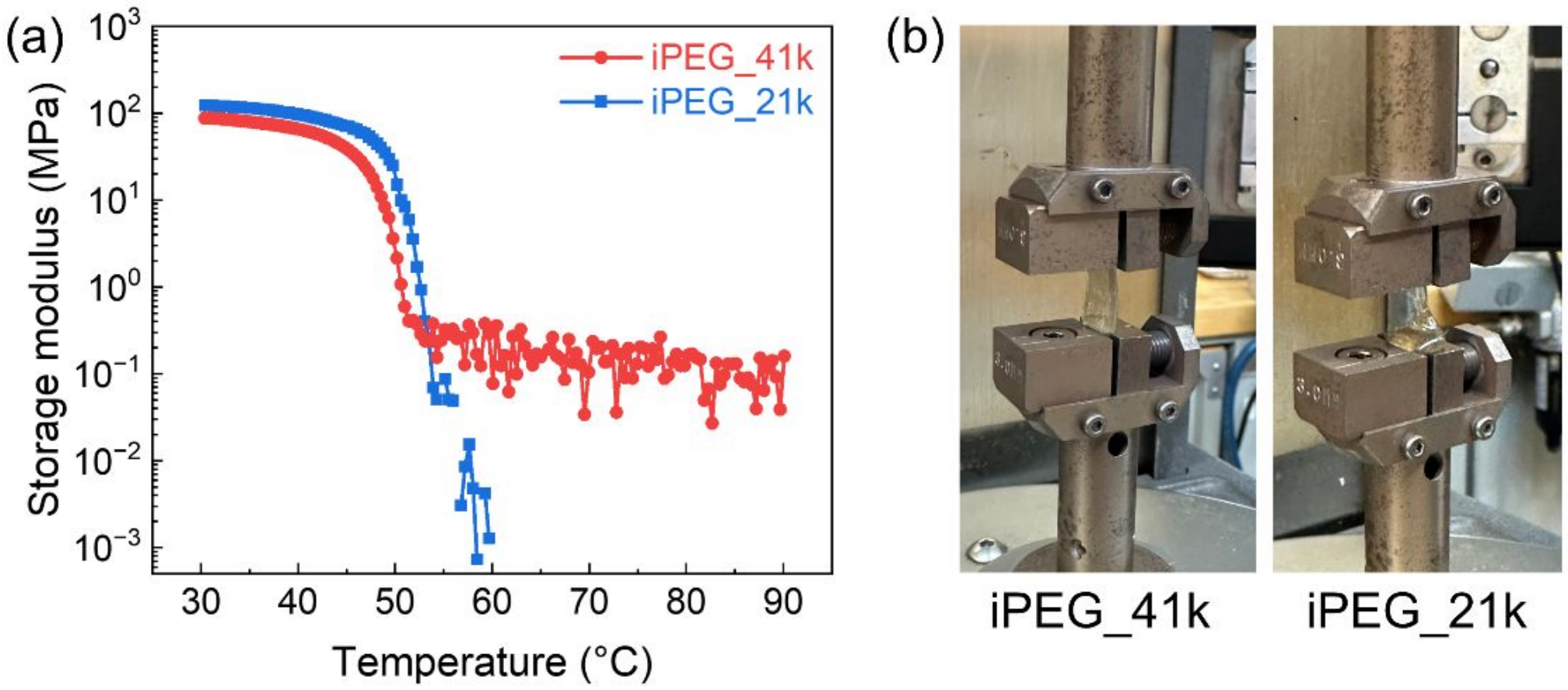

Figure S7. Comparison of the thermal stability of iPEG_41k and iPEG_21k. (a) Storage modulus (G') of iPEGs from 30 to 90 °C. (b) Photographs of samples immediately after the temperature ramp: iPEG_41k retains its original shape, while iPEG_21k undergoes significant deformation and flows as a viscous liquid.

2.5 Differential scanning calorimetry (DSC)

Melting temperature (T_m) and degree of crystallization (X_c) was determined by TA Instruments DSC 2500. Polymer samples (3−5 mg) were loaded in Tzero pans and sealed with Tzero lids. Experiments using the DSC 2500 involved two heating and one cooling cycles. A ramp rate of 10 °C/min was used for all heating cycles, and 20 °C/min was used for all cooling cycle. T_m was identified from the peak position of melting peak in the second heating cycle. X_c was calculated by evaluating the area under the melting peak in the first heating cycle, which corresponds to the enthalpy change (ΔH) during the melting transition. This value was compared against the theoretical heat of fusion for 100% crystalline PEG (ΔH_{cryst} = 197 J/g) using the formula (2)[2–4]:

$$X_C = \frac{\Delta H}{\Delta H_{cryst}} \cdot 100\% \quad (2)$$

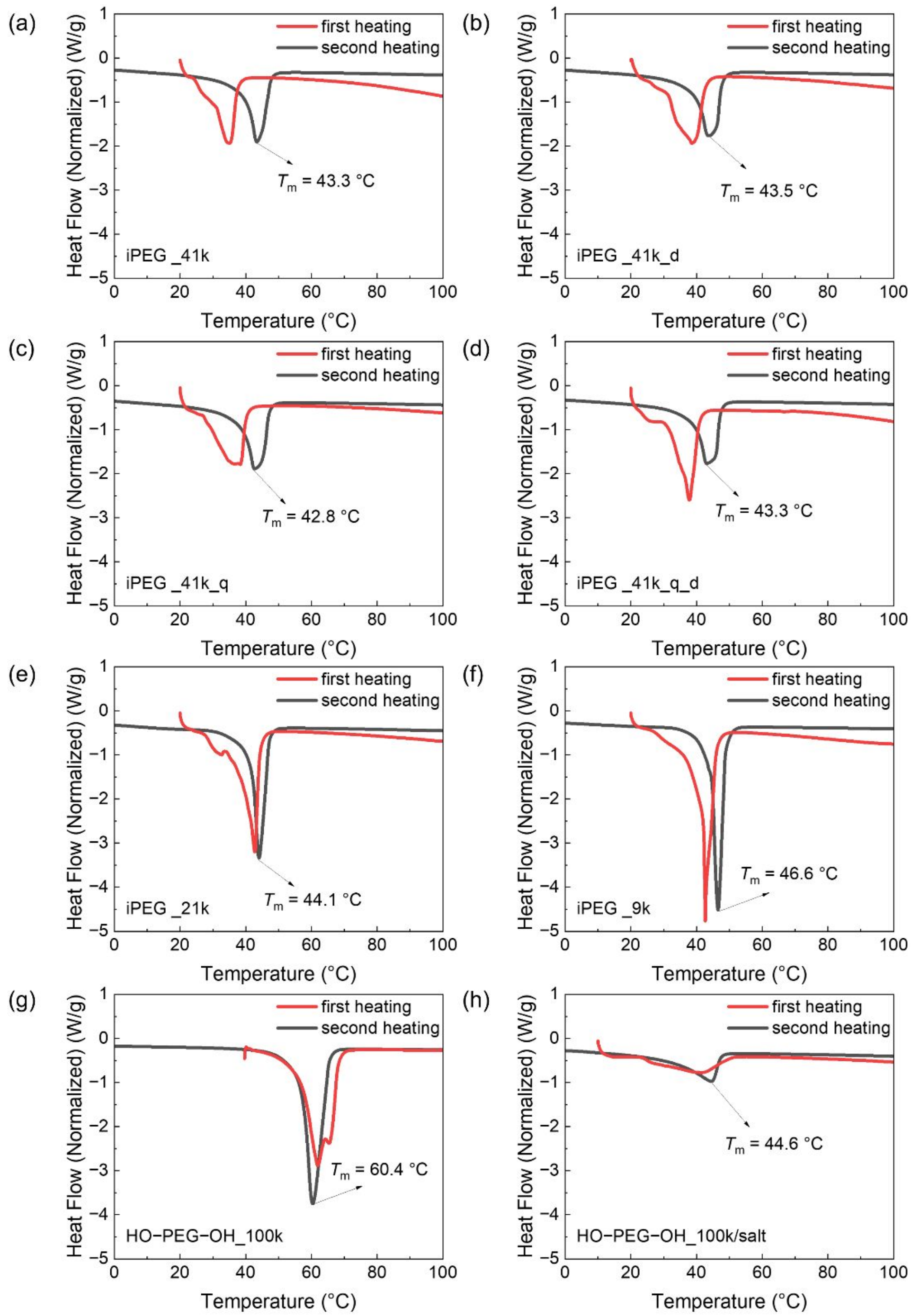

Figure S8. Differential scanning calorimetry thermograms of iPEGs and HO−PEG−OH.

2.6 Thermogravimetric analysis (TGA)

Thermogravimetric analysis (TGA) was performed on a TA Instruments TGA 5500. Samples (3−5 mg) were heated from room temperature to 100 °C at 20 °C/min, held isothermally for 10 minutes to remove absorbed moisture, and then heated to 600 °C at 10 °C/min. All measurements were conducted under a nitrogen purge of 25 mL/min. The water content was defined as the weight loss recorded at the end of the 10-minute isothermal step at 100 °C. Minimal weight change was observed during the isothermal hold itself, confirming that this step was sufficient for drying. The decomposition temperature (T_d) was defined as the temperature corresponding to 5% weight loss from the dried mass.

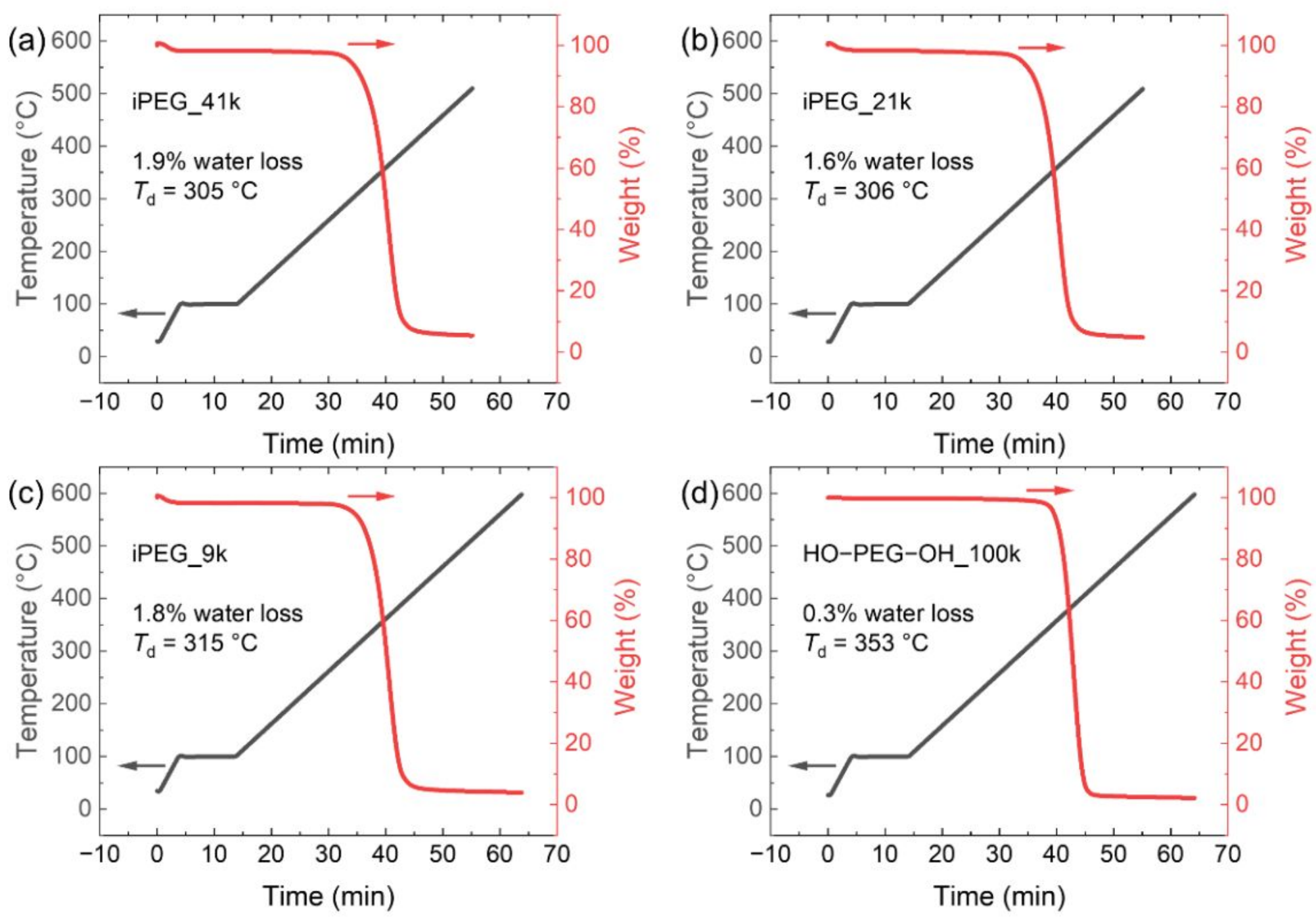

Figure S9. Thermogravimetric analysis (TGA) of iPEGs and HO−PEG−OH_100k showing low moisture content and thermal stability.

2.7 Small- and wide-angle X-ray scattering (SAXS/WAXS)

Small- and wide-angle X-ray scattering (SAXS/WAXS) data were obtained at Xenocs 3.0 system (Xenocs, Grenoble, France) at the Texas A&M University Soft Matter Facility. This instrument employs a GeniX 3D X-ray beam delivery system to generate monochromatic Cu Kα radiation (λ = 1.54 Å). Scattering patterns were collected using a Dectris Eiger 2R 1M-pixel 2D detector with sample-to-detector distance of 0.900 m for SAXS and 0.045 m for WAXS. Measurements were performed at room temperature. Samples were loaded in 316 stainless steel and sealed with Kapton tapes or directly load into the holder using the Kapton tapes. The 2D isotropic scattering patterns were reduced to give a 1D scattering intensity as a function of the wavevector $q = 4\pi \sin(\theta/2)/\lambda$, where θ is the scattering angle and λ is the wavelength.

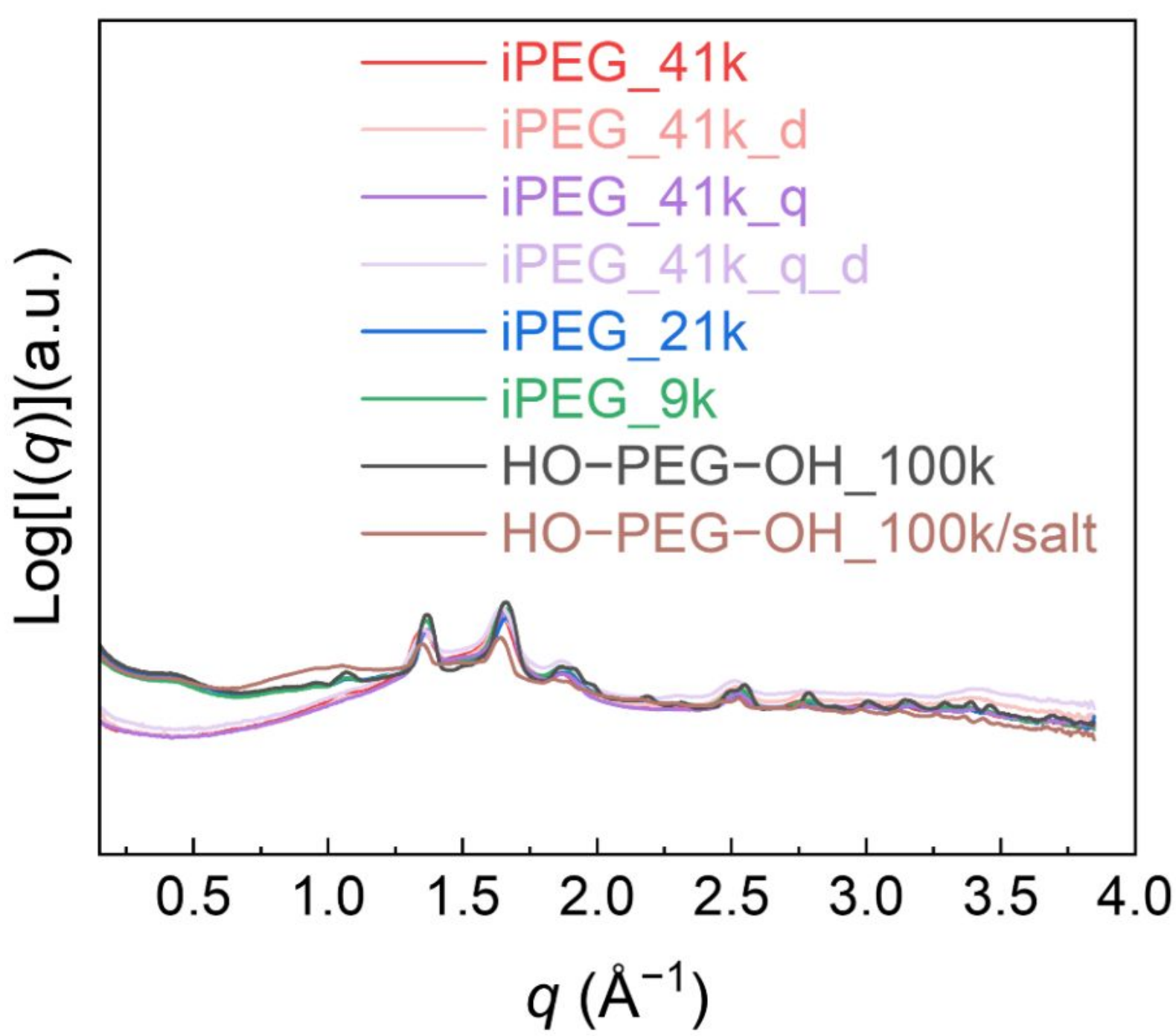

Figure S10. WXAS profiles of iPEGs and HO−PEG−OH_100k at room temperature.

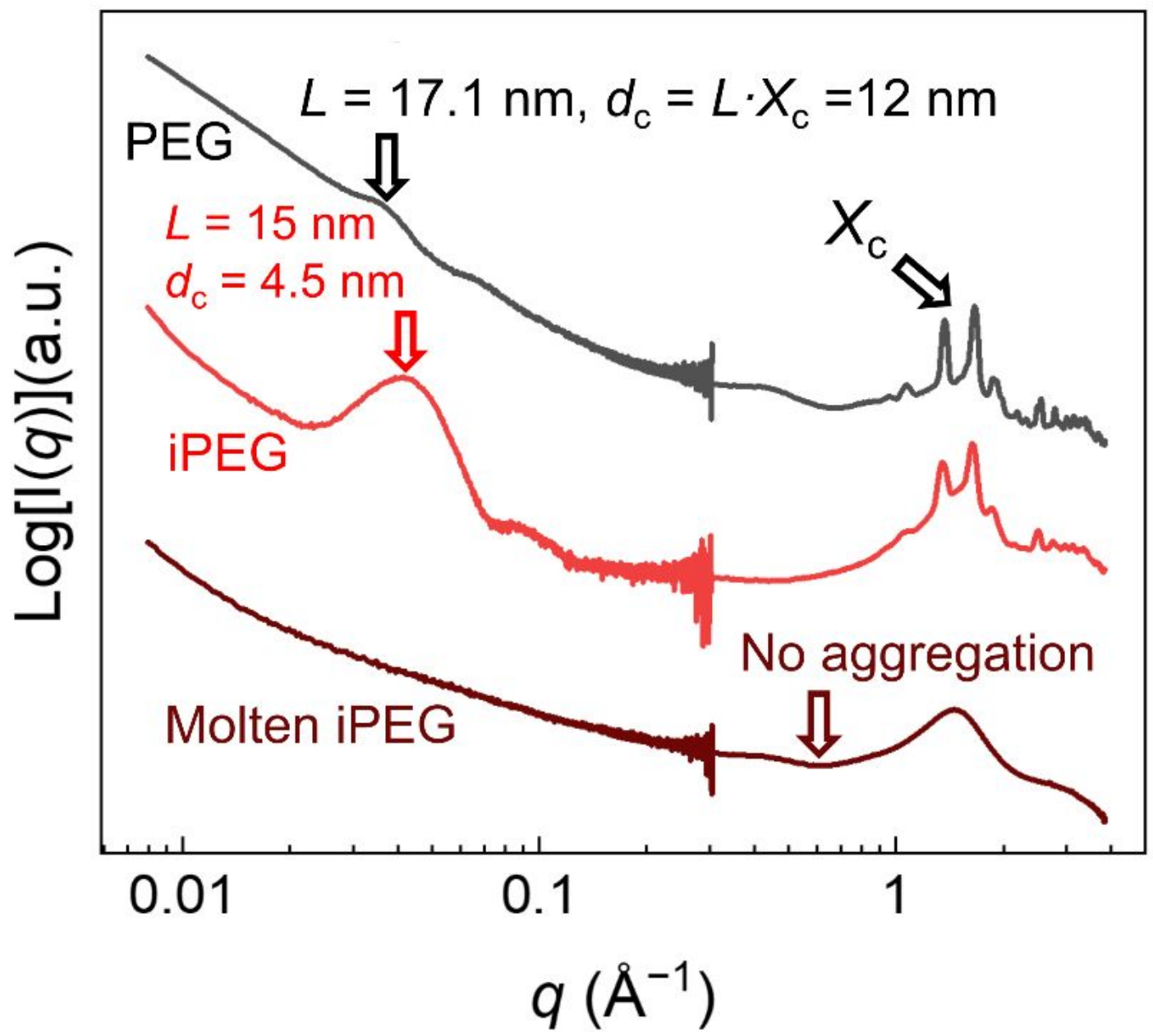

Figure S11. SAXS/WAXS profiles of HO−PEG−OH_100k, iPEG_41k, and molten iPEG_41k. Compared with HO−PEG−OH_100k, iPEG_41k exhibits a smaller long period and much thinner lamellae, consistent with suppressed lamellar thickening. In the melt, iPEG_41k shows no detectable scattering peak attributable to ionic aggregation, consistent with the absence of pronounced nanoscale ionic cluster formation.

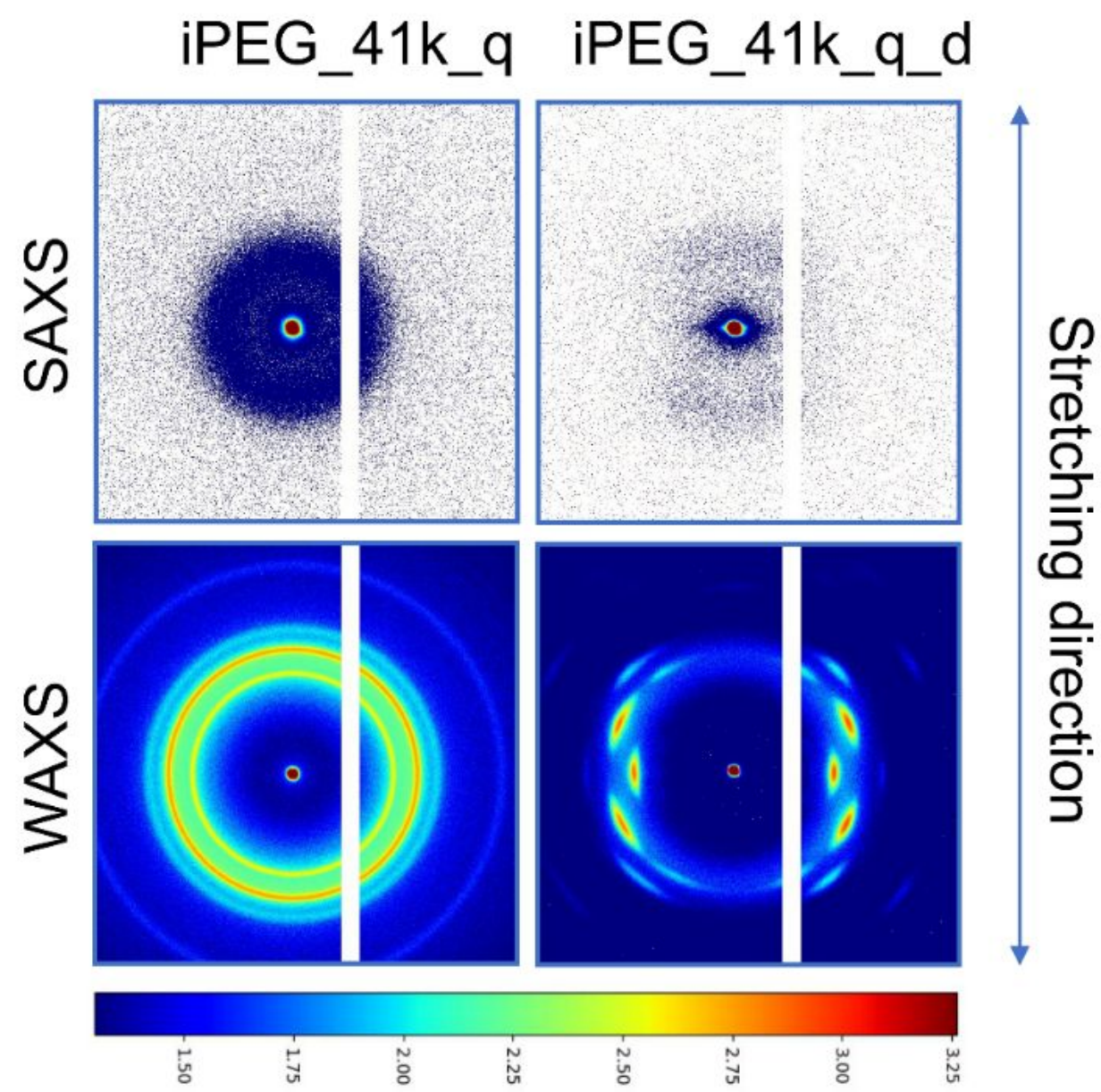

Figure S12. 2D patterns of LN quenched iPEG _41k (iPEG_41k_q) before and after stretching. For stretching sample (iPEG_41k_q_d): L = 10.3 nm, d_c = 4.0 nm, d_a = 6.3 nm (X_c = 39%).

3. Huang-Brown Model

The 1D Huang-Brown model[5–7] is employed to calculate the probability P that an amorphous polymer chain's end-to-end distance $(Nb^2)^{1/2}$ (assuming Gaussian coil) is greater than the critical distance $(2d_c + d_a)$, which a chain must span to form a tie between two crystalline lamellae.

$$P = \frac{\int_{2dc + da}^{\infty} dz\, z^2 exp(-\frac{3z^2}{2Nb^2})}{\int_{0}^{\infty} dz\, z^2 exp(-\frac{3z^2}{2Nb^2})} \tag{3}$$

where N is polymer's degree of polymerization, d_c is the crystal lamellar thickness, d_a is the amorphous thickness, b = 0.6 nm as the statistical segment length for PEG.[1]

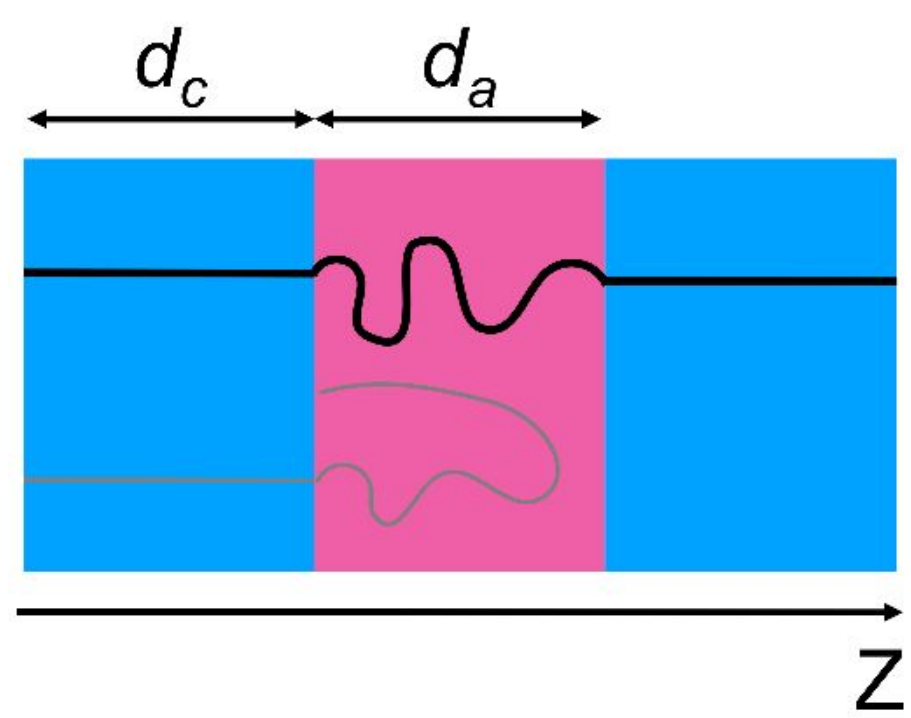

Figure S13. Schematic of interlamellar amorphous chain conformation along z direction.

4. References

(1) Lodge, T.; Hiemenz, P. C. *Polymer Chemistry*, Third edition.; CRC Press, Taylor & Francis Group: Boca Raton, 2020.

(2) Schulz, M.; Schäfer, M.; Saalwächter, K.; Thurn-Albrecht, T. Competition between Crystal Growth and Intracrystalline Chain Diffusion Determines the Lamellar Thickness in Semicrystalline Polymers. *Nat Commun* **2022**, *13* (1), 119.

(3) Buckley, C. P.; Kovacs, A. J. Melting Behaviour of Low Molecular Weight Poly (Ethylene-Oxide) Fractions. *Colloid & Polymer Sci* **1976**, *254* (8), 695–715.

(4) Golitsyn, Y.; Pulst, M.; Samiullah, M. H.; Busse, K.; Kressler, J.; Reichert, D. Crystallization in PEG Networks: The Importance of Network Topology and Chain Tilt in Crystals. *Polymer* **2019**, *165*, 72–82.

(5) Huang, Y.-L.; Brown, N. The Effect of Molecular Weight on Slow Crack Growth in Linear Polyethylene Homopolymers. *J Mater Sci* **1988**, *23* (10), 3648–3655.

(6) Huang, Y.-L.; Brown, N. Dependence of Slow Crack Growth in Polyethylene on Butyl Branch Density: Morphology and Theory. *Journal of Polymer Science Part B: Polymer Physics* **1991**, *29* (1), 129–137.

(7) Gu, K.; Snyder, C. R.; Onorato, J.; Luscombe, C. K.; Bosse, A. W.; Loo, Y.-L. Assessing the Huang–Brown Description of Tie Chains for Charge Transport in Conjugated Polymers. *ACS Macro Lett.* **2018**, *7* (11), 1333–1338.
